# SNP markers revealed the genetic diversity and population structure of *Mesosphaerum suaveolens* (L.) Kuntze Syn. *Hyptis suaveolens* (L.) Poit accessions collected in Benin

**DOI:** 10.1371/journal.pone.0331702

**Published:** 2025-09-04

**Authors:** Armel Frida Dossa, Dèdéou A. Tchokponhoué, Aristide Carlos Houdegbe, Enoch G. Achigan-Dako

**Affiliations:** Genetics, Biotechnology and Seed Science Unit (GBioS), Laboratory of Plant Production, Physiology and Plant Breeding (PAGEV), School of Plant Sciences, University of Abomey-Calavi, Abomey-Calavi, Republic of Benin; Museu Paraense Emilio Goeldi, BRAZIL

## Abstract

*Mesosphaerum suaveolens* (L.) Kuntze is a wild species with many biological activities in medicine. The species can potentially serve as a pesticide in agriculture thanks to its high content of volatile compounds. However, knowledge of the species’ genetic diversity and population structure is currently scarce, thus hindering improvement and conservation initiatives. This research used single nucleotide polymorphism (SNP) genotyping to study the diversity and population structure of 175 *Mesosphaerum suaveolens* collected from the three phytogeographical zones of Benin. Our study reports 3,613 high-quality SNP markers for *M. suaveolens.* Based on the markers, a mean polymorphism information content of 0.28, a low expected heterozygosity (He* *= 0.287) coupled with low genetic differentiation (Fst = 0.007) were observed in the species. The overall average value of observed heterozygosity (*Ho*) was 0.11. The Neighbor-joining analysis identified three subpopulations, whereas two mixed subpopulations were detected via population structure analysis. The results of the molecular variance analysis revealed that differences among samples within populations accounted for the majority of genetic variation (59.3%). The population from the Sudanian region presented the highest genetic diversity (He = 0.293), whereas the Guineo-congolian population presented the lowest genetic diversity (Ho = 0.278). These results pave the way for informed conservation in *M. suaveolens* and highlight the need to expand the genetic base of this species for upcoming improvement initiatives. However, reference genome and genome-wide association studies (GWAS) to associate genomic and morphological or chemical constituents are needed.

## 1. Introduction

Pignut [*Mesosphaerum suaveolens* (L.) Kuntze Syn. *Hyptis suaveolens* (L.) Poit] is a wild species of the Lamiaceae family that originates in the tropical and subtropical climate of America [1]. The species is a heterozygous polyploid with a chromosome number ranging from 2n = 28 to 2n = 32 [2]. This aromatic perennial species is spread worldwide, reaching all continents including Europe, Africa, Asia, and Oceania, and can reach 2 m in height [3,4]. The reproduction of pignut occurs through both self and cross-pollination (geitonogamy and xenogamy). Sexual reproduction facilitated by insect and wind, results in genetic variability within the species [5]. Sharma and Sharma [6] reported that changes from outcrossing to inbreeding contribute to the successful invasiveness of the species. Though considered invasive, *M. suaveolens* is a valuable species due to its essential oils which exhibit high medicinal and insecticidal properties.

Essential oils are environmentally-friendly alternatives to synthetic pesticides, making them a popular choice for pest management globally. The essential oils of *M. suaveolens* exhibit a broad range of biological activities against various storage and field pests thanks to the higher chemical variability of their essential oils. They have been shown to successfully control insects such as *Sitophilus zeamais* Motschulsky (Coleoptera: Curculionidae), *Maruca vitrata* Fabricius (Lepidoptera: Crambidae), *Megalurothrips sjostedti* Trybom (Thysanoptera: Thripidae), and *Aphis craccivora* Koch (Hemiptera: Aphididae) [7–9]. These findings position *M. suaveolens* as a promising aromatic plant for developing novel biological insecticides [10]. In addition to its pest control potential, *M. suaveolens* is considered as an eco-friendly herbicide due to its germination and seedling growth inhibition effect in weeds such as *Cereus jamacaru*. DC. Subsp. *jamacaru* (Cactaceae), and *Echinochloa crus-galli* (L.) P.Beauv. (Poaceae) associated with rice cultivation [11,12]. Beyond its agricultural applications, *M. suaveolens* has several medicinal properties [13]. Its roots, leaves, and twigs are used in decoctions to heal several ailments, including digestive infections, malaria, skin infections, constipation, renal inflammation, headaches, and respiratory issues injuries [8,14]. Additionally, the essential oils exhibit anti-cancer properties against human breast cancer, and possess anti-inflammatory properties [14–17]. Furthermore, the species possesses ecological value owing to its sturdy tolerance to drought and disturbed habitats [18].

Moreover, diversity in the chemical variability of the species essential oils was mentioned in numerous studies. Five *M. suaveolens*-chemotypes have been reported including 1,8-cineole and sabinene, eugenol and germacrene D, fenchone and limonene, sabinene and β-pinene and β-caryophyllene and 1,8-cineole [14,19–22]. In Benin, Salifou et al. [21] identified two of these chemotypes, namely, β-caryophyllene, and 1,8-cineole within all three phytogeographical regions (Guineo‒congolian, Sudano‒Guinean and Sudanian). This shows the potential for engaging in the species improvement to better exploit its richness.

Despite its potential, previous studies on *M. suaveolens* have focused predominantly on its chemical contents, biological activities against human and crop pathogens [20,23], morphological traits [24], reproductive and pollination ecology [5,6], and planting modes [25]. While its essential oil application as a biopesticide remains at the laboratory stage, commercialization is hindered by its low essential oil yield. Addressing these challenges requires agromorphological and genetic diversity studies to identify genotypes with higher yield and better oil quality, since variations in *M*. *suaveolens*’ essential oil quality and yield are linked to genotype and environment [10].

To date, information on the genome and genetic diversity structure of *M. suaveolens* is scarce. Meanwhile, other economically significant Lamiaceae species such as *Ocimum gratissimum* [26,27], and *Ocimum basilicum* [28,29], have undergone more detailed genomic studies. The increasing interest in *M. suaveolens*’ essential oil for agricultural and medicinal purposes, and lack of information on its genetics, highlight the need for genetic diversity studies to optimize its oil yield and quality.

**Fig 1:**
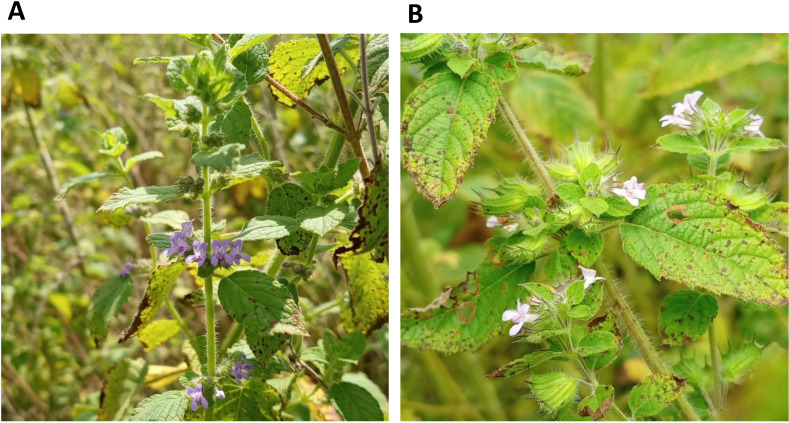
Inflorescence color-defined *Mesosphaerum suaveolens* morphotypes in Benin. (A): morphotype with a purple inflorescence; (B): morphotype with a light-purple inflorescence.

The key observations that predicted genetic diversity within the species include intrapopulation variations in seed and reproductive organs size [30]. Moreover, two morphological variants of *M. suaveolens*, white-flowering, and blue-flowering, have been reported in India and Brazil [31]. In Benin, empirical observations during fieldwork revealed a third variant with light violet flowers (Fig 1) in the Sudanian phytogeographical zone.

The first molecular evaluation of *M. suaveolens* diversity was conducted using intersimple sequence repeat (ISSR) markers in 2011 [31]. This study with ISSR primers (Hy1- Hy15) analyzed two flower color genotypes (blue and white), revealing distinct genetic basis between them. However, limitations, such as an unweighted degree of difference and limited samples and field observations reduced the study’s robustness [31]. Out of 43 polymorphic alleles, 23.6% were unique to the blue flowering form, and 11.1% were exclusive to the white flowering. This was the only one genetic diversity study conducted to evaluate the species’ diversity until now. Despite being highly polymorphic, ISSR markers have limitations for resolving geographical differentiation when few genetic markers are used [32]. Hence, it is essential to evaluate the genetic diversity within the species with a high-resolution and robust molecular markers like single nucleotide polymorphism (SNP). SNP assays are an appropriate alternative for diversity analysis in *M. suaveolens* because of their high frequency and availability within the genome, which make them an excellent markers for evaluating genetic diversity [33]. With their saliency, relatively low cost, and accuracy, SNP assays are becoming popular and evolving as first-order markers [34]. Genotyping by sequencing (GBS) facilitates the detection of thousands of SNP markers from many individuals via a reduced representation library [35]. One of the most affordable, simple, and effective genotyping-by-sequencing platforms is the Diversity Array Technology Sequencing (DArTseq) platform, which enables easy identification of genome-wide markers via restriction enzymes, as well as sequencing of restriction elements [36]. DArTseq technology generates a large pool of high-quality SNPs and SilicoDArT markers, even in species without reference genome. Furthermore, DArTseq is an excellent approach for genotyping a wide number of individuals [34,37,38]. It has been used to reveal the genetic diversity and population structure of 62 accessions of aromatic basil (*Ocimum basilicum* L.) from Lamiaceae family in Ethiopia [38]. Numerous molecular studies have used DArTseq to explore the genetic diversity, to identify markers of interest, and assess the relationship among species lines of several crops such as *Vigna unguiculata* (L) Walp. [37], *Colocasia esculenta* (L.) Schott [39], *Synsepalum dulcificum* (Schumah & Thonn.) Daniell [40], *Zea mays* L. [41], *Phaseolus vulgaris* L. [42], *Musa acuminata* Colla [43] and *Macrotyloma geocarpum* (Harms) Maréchal & Baudet [44].

Despite the widespread use of single nucleotide polymorphisms in genetic diversity studies, no research has yet reported the genetic diversity of *M. suaveolens* using SNP markers worldwide. This represents a significant gap that our study seeks to address. By generating SNP markers from *M. suaveolens*, we will make this species’ first sequences accessible and lay a good foundation for further application in studying the species’ evolutionary history, conservation, genetic mechanism regulating essential oil yield and quality, and breeding purposes. The aim of this research was to generate molecular information of *M. suaveolens* that will facilitate genetic improvement of *M. suaveolens* accessions for improved essential oil yield and quality. Specifically, we aimed to assess the extent of genetic diversity and population structure in *M. suaveolens* germplasm from Benin using high-density SNP markers. To achieve this, the study seeks to answer the following research questions: 1) what is the extent of genetic diversity in the *M. suaveolens* germplasm studied? 2) does geographical distribution influence the genetic diversity of a species? 3) what is the genetic structure of the collected accessions? We hypothesized that 1) the accessions of *M. suaveolens* from close geographical origins presented molecular similarities and 2) the *M. suaveolens* collection encompassed more than one genetic cluster.

## 2. Materials and methods

### 2.1. Leaf sampling strategy

A total of one hundred seventy-five pignut accessions were sampled and georeferenced (S1 Table) with the approval of the local authorities. The collection covered three populations corresponding to the phytogeographical regions of Benin (the Guineo-congolian, Sudano-guinean, and Sudanian regions) from December 2019 to March 2020, with at least a minimal distance of 10 m between two accessions.

The Guineo-congolian zone, located in the southern part of Benin, has a subequatorial climate. It is characterized by a bimodal season, with two rainy seasons and two dry seasons. The annual precipitation ranges from 900 to 1,400 mm. The majority of soils are ferralitic, clayey, sandy, hydromorphic, low-lying, and vertisols. The vegetation of this zone comprises a mosaic of patches of dense rainforest, mangrove swamps, grasslands, savannahs, and fallow land [45]. The Sudano-guinean zone is the transition region. It is characterized by a unimodal season with an average rainfall ranging from 900 to 1,110 mm. The mineral soils here are not particularly well-developed or fertile, and the ferruginous soils on crystalline bedrock are variable in terms of fertility. The vegetation consists of wooded and shrubby savannahs, open forests, dense dry forests, and forest galleries. The Sudanian zone is distinguished by unimodal rainfall regime. The average annual precipitation ranges from 900 to 1,100 mm. The soils include hydromorphic, ferrallitic, lithosols, and drained soils (61,63).

A total of 93 accessions were sampled from the Guineo-congolian population, 46 from the Sudano-guinean population, and 36 from the Sudanian population. For each accession, 10 pest-free young leaves were sampled and automatically silica gel-dried [40] before being transported to the laboratory [Genetics, Biotechnology and Seed Science Unit (GBioS) in Abomey-Calavi]. The labelled accessions were then arranged in kits of 96 tubes each and shipped to SEQART AFRICA at the International Livestock Research Institute (ILRI) for genotyping.

### 2.2. National and local approval

Before embarking on the fieldwork to collect accessions, the first author secured the approval of the academic committee of the École Doctorale des Sciences Agronomiques et de l’Eau (EDSAE) of the Faculty of Agricultural Sciences (FSA) of the University of Abomey-Calavi (UAC). The ethics committee that waived the study was the first author’s thesis committee at the University of Abomey-Calavi, comprising Prof. Enoch G. Achigan-Dako, Prof. Eleonore Yayi-Ladekan, Prof. Bokonon-Ganta Aimee, Prof. Latifou Lagnikan, and Prof. Victorien T. Dougnon. The collection of the plant materials adhered to both the National and International Code of Conduct for Plant Germplasm collection. Prior to collecting samples, the objectives of the research project were presented to the local authorities of each village under investigation. The informed verbal consent of all local authorities was obtained before sample collection.

### 2.3. DNA extraction and sequencing

The extraction of DNA was performed with a Nucleomag Tissue Kit (DArtseq proprietary kit). The amount of isolated genomic DNA ranged between 50 and 100 ng/µl. On 0.8% agarose, the quantity and purity of the DNA were examined [42,44]. Libraries were constructed following Kilian et al. [46]. The genome complexity reduction approach used was that of Diversity Arrays Technology and Sequencing (DArTseq), whereby PstI and MseI enzymes were combined to digest the genomic DNA and then to ligate barcoded and common adapters [47]. The constructed libraries were sequenced via 77-base single-read sequencing runs. Next-generation sequencing was performed via HiSeq 2500, and DArTsoft14 was used to score the DArTseq SNP markers. The markers were scored as 0 (reference homozygous), 1 (homozygous), or 2 (heterozygous). The genomic matrix obtained was preliminarily quality-assessed with the “dartR package” [48] in the R environment (version 4.1.2). Only SNPs with a call rate *> *85%, a minor allele frequency (MAF) > 0.05, and a reproducibility rate > 95% were used [40,49] to constitute the genomic matrix of the 175 *M. suaveolens* accessions. Missing values of that genomic matrix were imputed on the KD computational platform (https://kdcompute.seqart.net/kdcompute/plugins) via the Ensemble Method algorithm to obtain a full genomic matrix for the analysis described below.

### 2.4. Genetic diversity analysis

First, the quality of the retained markers was explored through analysis of their polymorphic information content (PIC). The following overall genetic diversity indices were subsequently computed: expected heterozygosity (*He*), observed heterozygosity (*Ho*), total gene diversity (*Ht*), and the inbreeding coefficient (*F*_*IS*_). They were all estimated with the “dartR package” [48] in R (version 4.1.0). To determine the genetic diversity among populations, *He*, *Ho*, allelic richness, and *F*_*IS*_ were calculated across populations. To ascertain the existence of private alleles, the “poppr package” [50] was used. The overall differentiation index (Fstg) was calculated to assess the genetic differentiation within the studied germplasm, whereas pairwise genetic differentiation indices (Fstp) were computed between pairs of populations to understand the level of divergence between them [51].

### 2.5. Population structure

The population structure was assessed via three methods. First, an analysis of molecular variance was performed in the R environment (version 4.1.0) to estimate the relative contribution of variation sources (among populations, among samples within populations, within samples) to genetic variation in the species [39]. Second, STRUCTURE software version 2.3.4 [52] was used to implement Bayesian model clustering. Hypothetical number of clusters (K) ranged from 2 to 10, with an initial burn-in period of 10,000 for each run of 10,000 Markov Chain Monte Carlo (MCMC) iterations. The ΔK method was used to determine the highest predicted value of clusters (K) for each test via the online program Structure Harvester [53,54]. The average silhouette method was used at the appropriate K-value to determine the distribution of likelihood values of K, which exhibits a notable inflection point that was discernible in the Delta K-values. Only accessions with a membership coefficient ≥ 0.7 for a cluster were classified as belonging to that cluster [55]. Consequently, accessions with a membership coefficient < 0.70 at every given K were categorized under the admixture group. Lastly, the relationships among the 175 *M. suaveolens*, a phylogenetic tree, were assessed via neighbor-joining tree analysis via Molecular Evolutionary Genetics Analysis X (MEGAX) software [56] based on the maximum composite likelihood evolutionary distance. Visualization was performed via the interactive tree of life (ITOL, V.5) [57] web application (https://itol.embl.de/).

## 3. Results

### 3.1. Markers quality

Among the 18,205 raw SNP markers generated, 3,613 were ultimately retained after the quality assessment. These 3,613 markers have average polymorphism information content (PIC) values ranging from 0.09 to 0.49, with a mean value of 0.28 ± 0.002. The mutations noted in the DNA sequences are transitions (59.5%) and transversions (40.5%), with a transition/transversion rate of 1.5%. The proportions of C/T and A/G transitions were 31.4% and 28.1%, respectively. The proportions of A/T, A/C, G/T, and G/C transversions were 11.4%, 10.4%, 9.7%, and 8.4%, respectively. Minor allele frequency had an average value of 0.2 ± 0.1 and varied from 0.05 to 0.5.

### 3.2. Genetic diversity in *M. suaveolens* populations

The overall average value of observed heterozygosity (*Ho*) was 0.117, whereas the total gene diversity (*Ht*), expected heterozygosity (*He*), inbreeding coefficient (*F*_IS_), and interpopulation diversity (*D*_*ST*_) in the species were 0.289, 0.287, 0.592, and 0.002, respectively. The estimates of these statistics for each studied population are presented in Table 1. While expected heterozygosity estimates increased following the south-north ecological gradient, neither the observed nor the allelic richness followed this trend. Although the mean allelic richness seemed not statistically different among population, the Guinean population presented the highest value, whereas the Sudano-guinean population presented the lowest value for this parameter. For the observed heterozygosity, the Sudano-guinean population had the highest estimate (0.135), with the lowest level of inbreeding.

**Table 1:** Diversity statistics for the 175 *Mesosphaerum suaveolens* using 3,613 DArTseq-based single nucleotide polymorphism (SNP) data sets.

Population	*Ho*.adj	u*He*	*F*_IS_	Allelic richness
Guineo-congolian	0.104	0.278	0.625	1.991
Sudano-guinean	0.135	0.284	0.523	1.985
Sudanian	0.111	0.293	0.618	1.986
**Overall average values**	**0.117**	**0.287**	**0.592**	

*Ho*.adj, observed heterozygosity; *He*, expected heterozygosity; *F*_IS_, inbreeding coefficient.

No private alleles were detected among the three populations. However, our analysis identified one allele that was specific to the Sudanian and Sudano-guinean populations when compared to the Guineo-congolian population. The set of Guineo-congolian and Sudano-guinean populations shared 27 private alleles by comparing them with the Sudanian population. Similarly, 20 alleles were specific to the Guineo-congolian and Sudano-guinean populations when compared with the Sudanian population (Table 2).

**Table 2:** Alleles privacy among groups of sampled populations.

Comparison between Pop1 and Pop2		Priv1	Priv2	Total priv	AFD
Pop1	Pop2				
Guineo-congolian (GC)	S + SG	0	1	1	0.059
Sudano-guinean (SG)	GC + S	0	20	20	0.050
Sudanian (S)	GC + SG	0	27	27	0.068

GC, Guineo-congolian; SG, Sudano-guinean; S, Sudanian; Pop1, reference population; Pop2, All other population except the reference population; AFD, Allelic fixed difference; Priv 1, Reference population’s Private allele; Priv 2, Private allele of all other population except the reference population.

### 3.3. Genetic differentiation

The overall genetic fixation index (Fstg) value for *Mesosphaerum suaveolens* was quasi-null (Fstg = 0.007), suggesting very low differentiation trend in the species. The pairwise differentiation indices between the pairs of studied populations are shown in Table 3. The greatest pairwise differentiation index (Fstp = 0.018) was observed between the Sudanian and Guineo-congolian populations, whereas the lowest (Fstp = 0.006) was recorded between the Sudano-guinean and Sudanian populations (Table 3).

**Table 3:** Pairwise Cockerman genetic fixation index (Fst) values between *Mesosphaerum suaveolens* populations.

Populations	Guineo-congolian	Sudano-guinean
**Sudano-guinean**	0.007	
**Sudanian**	0.018	0.006

### 3.4. Population structure

The results of the hierarchical analysis of molecular variance (AMOVA) revealed that the genetic variation observed was mainly due to variation among samples within populations (59.3%), whereas variation within samples accounted for 39.6% of the total genetic variation (Table 4). Only 1.1% of the molecular variation was due to variation among populations (phytogeographical regions).

**Table 4:** Analysis of molecular variance (AMOVA) based on three phytogeographical regions (Guineo-congolian, Sudano-guinean, and Sudanian), 175 individual accessions, and 3,613 single nucleotide polymorphism (SNP) markers.

Source of variation	df	Sum of Squares	Mean Squares	% Variation	P-value
Among populations	2	2866.77	1433.38	1.1	< 0.001
Among samples within populations	172	141573.92	823.10	59.38	< 0.001
Within samples	175	36072.5	206.12	39.6	< 0.001
Total	349	180513.19	517.22	100.0	

df = degrees of freedom.

**Fig 2:**
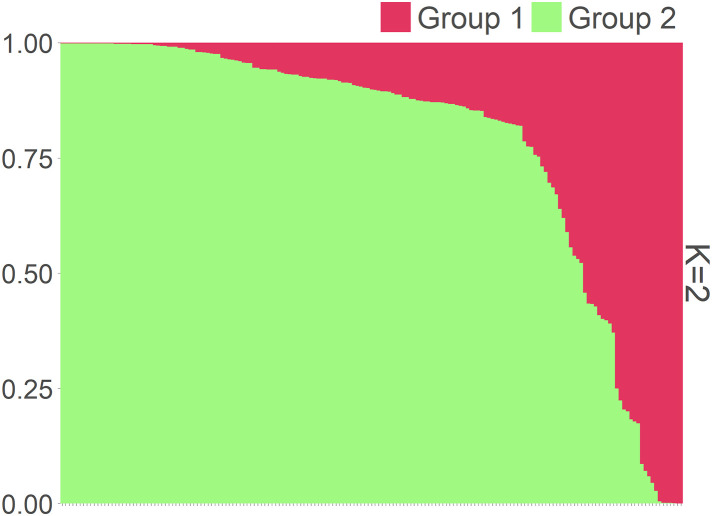
Barplot showing the structuring pattern of the 175 *Mesosphaerum suaveolens* accessions.

The population structure inferred from the Bayesian clustering approach suggested an optimal value of K = 2 (S1 Fig), thus defining two clusters for the 175 accessions (Fig 2). Based on membership probabilities of ≥ 0.70 (S2 Table), cluster 1 (C1) comprises 18 accessions, and cluster 2 (C2) comprises 140 accessions. The remaining 17 accessions were placed in the admixture group. Cluster 1 (C1) encompassed individuals from all regions, with 5.5% belonging to Sudanian, 5.5% belonging to Sudano-guinean, and 89% belonging to Guineo-congolian regions. Cluster 2 (C2) comprised 48.6% Guineo-congolian accessions. Guineo-congolian accessions (58.8%) also largely dominated the admixture group.

**Fig 3:**
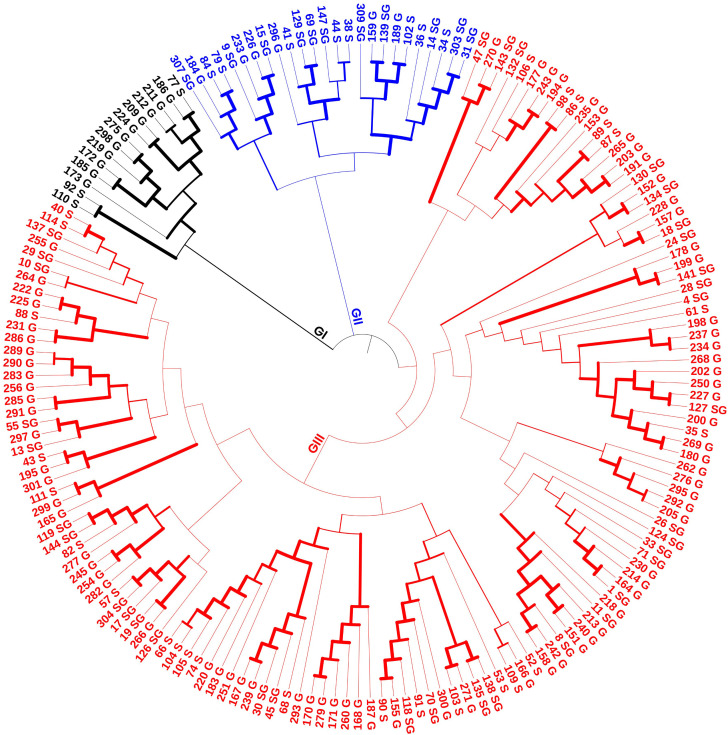
Neighbor-joining tree depicting the relationship among the 175 *Mesosphaerum suaveolens* accessions based on 3,613 single nucleotide polymorphism (SNP) markers. Group I (Color black), Group II (Colour Blue), and Group III (Color red).

The accessions were structured based on the neighbor-joining tree, and the 175 accessions were classified into three groups: group I, group II, and group III. Group I, consists of 14 accessions. Group II includes 25 accessions, and group III comprises 136 accessions (77.8%). The largest group III includes some individuals from cluster 2 (C2), along with all accessions in cluster 1 (C1) identified by STRUCTURE, as well as all admixed accessions (Fig 3).

## 4. Discussion

The present study is the first to depict the extent of genetic diversity in pignut (*M. suaveolens*) using DArTseq technology with 3,613 high-quality SNP markers. This generates basic molecular information about *M. suaveolens* for future research linked to conservation and breeding. Among the identified SNP variants, A/G and C/T were the most common SNP variants in the studied *M. suaveolens* accessions. This finding is consistent with previous reports in both wild (*Amorphophallus paeoniifolius*) [58] and cultivated species (*Vigna unguiculata* and *Musa acuminata* cv. Sotoumon) [37,43,55], where transitions were the common source of genetic variation. In this study, the retained SNPs were moderately informative, as the Polymorphism Information Content (PIC) values fell within the range of 0.25–0.5 [59]. This informativeness is similar to values reported in an Ethiopian *Ocimum basilicum* study, which varied from 0.23 to 0.37 using DArTseq [38]. Overall, the markers used were informative enough to help appreciate the extent of diversity in the species. The moderate PIC value of the SNP markers in this study may be enhanced by a large number of markers and a wide genome coverage [42].

The average expected heterozygosity (*He*) was 0.287, indicating low genetic diversity in the studied *M. suaveolens* germplasm. This result corroborates the findings of Gossa et al. [38], where *O*. *basilicum* populations exhibited low diversity (*He* varied from 0.13 to 0.33) when evaluated using SNP markers. Similarly, Kyrkjeeide et al. [60] reported comparable expected heterozygosity values (*He *= 0.27) for *Dracocephalum ruyschiana* accessions, an aromatic plant from the Lamiaceae family [60]. Low to moderate diversity was also observed in *Hyptis pectinata*, a close relative of *M. suaveolens* in Brazil using ISSR markers [59]. Given that widely distributed wild species such as *M. suaveolens* typically exhibit a relatively high level of diversity, the unexpected low genetic diversity observed in this study may be attributed to various factors. First, the geographical coverage of the study, although encompassing three demarcated ecological regions of Benin, is still limited to the Dahomey Gap, known as a single ecological area in the African rainforest space. We then speculated that expanding the collection areas to other countries or African ecological regions could potentially increase the diversity level [61]. Therefore, efforts must be undertaken to collect and preserve the genetic diversity of *M. suaveolens* present in Benin and worldwide. Second, the species was previously reported to exhibit both autogamy and allogamy, and the low diversity observed might indicate the preponderance of autogamy in the species, as was experimentally confirmed by Aluri and Reddi [62]. In addition, *M. suaveolens* species is facing landscape fragmentation in Benin, a phenomenon known to reduce the effective size of wild populations and, in turn limits genetic variation [58]. Given this phenomenon, the assessment of genetic diversity is fundamental to select worthwhile genotypes for future biological research studies. Furthermore, the populations’ size involved in this study could explain the low diversity observed, as it is reported that genetic diversity increases significantly as population size grows [63].

The low diversity in the present study, coupled with a moderate inbreeding coefficient (*F*_IS_ = 0.592), indicates a moderate level of fixation. This value of inbreeding could also justify the presence of both autogamy and allogamy in the species [5]. Among the studied populations, the Sudanian population exhibited the highest diversity, suggesting that it may have more potential to adapt to the changing climate in a region already known to experience severe climatic conditions. Therefore, this population is a potential source of genes of interest that could be deployed in improvement programs to develop specific product profile genotypes. Hence, there is an urgent need for more concentrated actions to collect genetic resources throughout the region for ex-situ conservation strategy profiling. Using SNP markers allowed an evaluation of the relationships for new future breeding or conservation efforts. The fixation index (Fst) measures a population’s differentiation linked to genetic structure [64]. An Fst value > 0.25 indicates very high differentiation, and an Fst < 0.05 implies low differentiation [65]. The values ranging from 0.05 to 0.15 and 0.15 to 0.25 reflected moderate and high differentiation, respectively. The Fst value (Fstg = 0.007) observed in this study indicates very low genetic differentiation between these three populations. Similarly, a low genetic differentiation among populations was reported for many other species from the Lamiaceae family, including *Dracocephalum ruyschiana* and *Dracocephalum austriacum* [66]. The very low differentiation observed in this study may also be associated with regions of samples collection or distance among the studied populations. As shown in Table 3, the closer the populations are, the smaller their genetic distances are, and the further apart they are, the greater their genetic distances are. The Fst value also indicates high gene flow among populations. Gene flow, which enhances genetic diversity within populations and reduces diversity among populations, is a factor affecting the genetic differentiation of populations, contrary to the effect of genetic drift [67]. Yao et al. [68] observed low differentiation in cowpea (*Vigna unguiculata*) populations due to long-distance gene dispersal, either by pollen or by seed. This mechanism could also explain the low differentiation noted in this study, as the sexual reproduction in *M. suaveolens* is possible through wind and insects contribution [62]. However, the current very low differentiation between *M. suaveolens* populations is contrasting the findings of Salifou et al. [21] based on the chemical constituents of the essential oil. Salifou et al. [21] reported two chemotypes of *M. suaveolens* according to the three phytogeographical regions of Benin, β-caryophyllene (Sudanian and Sudano-guinean), and 1,8-cineole (Guineo-congolian), which is somewhat predictive of genotype grouping based on accessions’ location. The accumulation of bioactive compounds in *M. suaveolens* varies across different regions due to environmental factors, genetic diversity, elicitors, and ecological conditions [17,69,70]. Studies highlighted that elements like soil composition, climate, and exposure to biotic and abiotic stress influence the production of secondary metabolites in this species [71,72]. The low diversity coupled with very low differentiation observed in this study suggests that, beyond genetic factors, environmental factors are key elements to be considered in trait enhancement, particularly in the improvement of the essential oils, which is the primary focus for *M. suaveolens*. This finding further highlights the need to expand the collection of accessions to access greater genetic variability. Selecting genotypes that remain stable across different environments in terms of yield and essential oil composition is therefore vital, reinforcing the importance of integrating genetic data with the chemical profiling of essential oils.

Differences in population structure display genetic variation and highlight a species’ capability to adapt to a changing habitat [73]. The neighbor-joining analysis detected three clusters, whereas the STRUCTURE analysis revealed two genetic groups. This difference can be partly explained by the restriction of the software STRUCTURE to accurately handle population structuring [44]. In addition to this limitation, the results of STRUCTURE are influenced by factors such as markers type, the number of studied populations, sample size, and number of loci scored [54]. Similar mismatching between neighbor-joining and structure analysis have been reported by Akohoué et al. [44] and Tchokponhoué et al. [40] while structuring Kersting’s groundnut and the Sisrè berry populations, respectively. Moreover, the Bayesian STRUCTURE analysis identified two groups, and 9.7% of the 175 *M. suaveolens* accessions were admixed. Pyne et al. [74] reported a 15.6% admixture among 180 *Ocimum* spp. accessions studied. This level of admixture could further support the assumption of genomic content exchange, possibly during cross-pollination [74]. This phenomenon has also been observed in *Elsholtzia stauntonii* Benth. (Lamiaceae) [61]. The results of this study indicate that natural outcrossing has preserved genetic diversity within *M. suaveolens* accessions. Furthermore, some accessions collected in the same districts were structured into different clusters, indicating that natural selection, genetic mutation, and migration could be the main mechanisms of evolution that affect these genetic differences [75]. According to the neighbor-joining tree, 77.8% of the sampled accessions were grouped into a single cluster which included both blue flowering and light purple flowering *M. suaveolens*). The accessions in this group were collected from diverse habitats, including mountainous region, easy-to-access areas, and abandoned fields [76], where anthropic activities and animals are permanent. The species’ habitat was mostly found along roadside. *M. suaveolens* produces abundant, lightweight, and small seeds that can be easily transported without difficulty by water and wind over long distances. This could explain the high gene flow observed among populations and accessions. The genetic similarity among accessions further supports this hypothesis.

To fully understand the population divergence of *M. suaveolens*, extensive germplasm collection across different regions of the world is necessary. Considering the low genetic diversity within *M. suaveolens* and the very low differentiation among populations, broadening the genetic base within the species could be helpful for future improvement programs. This will require cross-pollination. Cross-pollination increases genetic diversity through introducing new alleles and increasing variation [63]. In the current case, cross-pollination could enhance the genetic diversity by crossing the studied populations with another genetically distant population. Thus, introducing new germplasms and facilitating crosses between genotypes from different continents and countries would be essential steps toward to increase the genetic diversity of *M. suaveolens*.

## 5. Conclusion

This SNP-based characterization study generated the first wide genetic polymorphism information in *M. suaveolens*. A total of 3,613 SNP markers with minor allele frequency ≥ 0.05 were discovered. A low genetic diversity within *M. suaveolens* accessions and a very low differentiation among studied populations were observed. Two distinct genetic clusters were identified in the collected accessions; yet the genetic diversity was not structured according to phytogeographical regions. Among the studied populations, the Sudanian population exhibited higher diversity than the Guineo-congolian and Guineo-sudanian populations. The discovered DArTseq SNP markers represent a novel genomic resources to improve *M. suaveolens* population management and breeding program. These resources could aid in improving traits like essential oil yield and quality, and the biosynthesis of bioactive compounds. The findings open rooms for the genome assembly of *M. suaveolens* and further research combining morphological, chemical constituents, and molecular analysis to allow genome-wide association studies. Worldwide, this study is the first to describe the population structure and genetic diversity in *M. suaveolens* using SNP, thus setting the scene for a proper marker-assisted improvement of useful traits in the species.

## Supporting information

S1 Table Coordinates for each accession from the three phytogeographical regions of Benin. This is a word file presenting the coordinates of each accession sampled.
(DOCX)

S2 Table *Mesosphaerum suaveolens* accessions grouping based of the membership value (Q). Admixt = Admixed individuals, C1 = Individuals of the group 1 and C2 = Individuals of the group 2. This is a word file presenting the accessions grouping.
(DOCX)

S1 Fig Estimate of the optimal number of clusters with K ranging from 1 to 10. (PDF)

## References

[1] Ogunmefun OT, Obi PU, Akpor OB. Bioinsecticidal efficacy of *Eucalyptus camaldulensis* (Dehn) and *Hyptis suaveolens* (L.) Poit. leaf extracts on *Callosobruchus maculatus* (Cowpea weevil). Sci Afr. 2023;20:e01663. doi: 10.1016/j.sciaf.2023.e01663

[2] Coleman JR. Chromosome-numbers of angiosperms collected in the state of Sao-paulo. Revista Brasileira de Genética. 1982;5:533–49.

[3] Lima AS, Fernandes YML, Silva CR, Costa-Junior LM, Figueiredo PLB, Monteiro OS, et al. Anthelmintic evaluation and essential oils composition of Hyptis dilatata Benth. and Mesosphaerum suaveolens Kuntze from the Brazilian Amazon. Acta Trop. 2022;228:106321. doi: 10.1016/j.actatropica.2022.106321 35063413

[4] Saha PR, Sinha S, Sinha RK. Morphometrical diversity of reproductive structures in *Hyptis suaveolens* (L.) Poit. : an ethnomedicinal weed of Lamiaceae. Curr Bot. 2017;8. doi: 10.19071/cb.2017.v8.3200

[5] Aluri RJ, Reddi SC. The explosive floral-mechanism and pollination in the genus *Hyptis* (Lamiaceae). Proceedings-Indian National Science Academy Part B. 1996;62:117–24.

[6] Sharma P, Sharma N. Mating strategies in a ruderal weed: case history of *Hyptis suaveolens* (L.) Poit. Vegetos. 2019;32(4):564–70. doi: 10.1007/s42535-019-00070-z

[7] Noudegbessi AM, Alabi OY, Sikirou R. Olfactory responses of *Sitophilus zeamais* L. to bushmint leaf powder and methanol extract on stored maize. Afr Crop Sci J. 1970;29(1):1–12. doi: 10.4314/acsj.v29i1.1

[8] dos Santos KP, Sedano-Partida MD, Sala-Carvalho WR, Loureiro BOSJ, da Silva-Luz CL, Furlan CM. Biological activity of *Hyptis* Jacq. (Lamiaceae) is determined by the environment. Ind Crops Prod. 2018;112:705–15. doi: 10.1016/j.indcrop.2017.12.065

[9] Pokhrel B, Choden D. Antifungal efficacy of *Hyptis suaveolens* and Rumex nepalensis extracts against *Alternaria solani*: An approach for bio-pesticides. Biocatal Agric Biotechnol. 2022;43:102439. doi: 10.1016/j.bcab.2022.102439

[10] Dossa AF, Fassinou Hotegni NV, N’Danikou S, Yayi-Ladekan E, Adjé CAO, Lagnika L, et al. Genetic diversity, essential oil’s chemical constituents of aromatic plant *Mesosphaerum suaveolens* (L.) Kuntze Syn. *Hyptis suaveolens* (L.) Poit. and its uses in crop protection: a review. Front Plant Sci. 2024;15:1454146. doi: 10.3389/fpls.2024.1454146 39363920 PMC11446852

[11] Bezerra JWA, dos Santos MAF, Meiado MV, Linhares KV, Boligon AA, dos Leandro CS, et al. Allelopathy of Aromatic Species on the Germination of *Cereus jamacaru* DC. subsp. *jamacaru* (Cactaceae). J Agric Sci. 2018;10(11):337. doi: 10.5539/jas.v10n11p337

[12] Sharma A, Singh HP, Batish DR, Kohli RK. Chemical profiling, cytotoxicity and phytotoxicity of foliar volatiles of *Hyptis suaveolens*. Ecotoxicol Environ Saf. 2019;171:863–70. doi: 10.1016/j.ecoenv.2018.12.091 30665103

[13] Nebié B, Dabiré CM, Bationo RK, Soma DD, Namountougou M, Sosso S, et al. Investigation on chemical composition and insecticidal activity against *Anopheles gambiae* of essential oil obtained by co-distillation of *Cymbopogon citratus* and *Hyptis suaveolens* from Western Burkina Faso. Malar J. 2024;23(1):339. doi: 10.1186/s12936-024-05177-6 39529037 PMC11552162

[14] Mishra P, Sohrab S, Mishra SK. A review on the phytochemical and pharmacological properties of *Hyptis suaveolens* (L.) Poit. Futur J Pharm Sci. 2021;7(1):65. doi: 10.1186/s43094-021-00219-1 33728353 PMC7952497

[15] Mohanta O, Ray A, Jena S, Sahoo A, Panda SS, Das PK, et al. *Mesosphaerum suaveolens* Essential Oil Attenuates Inflammatory Response and Oxidative Stress in LPS-Stimulated RAW 264.7 Macrophages by Regulating NF-κB Signaling Pathway. Molecules. 2023;28(15):5817. doi: 10.3390/molecules28155817 37570786 PMC10420984

[16] Bayala B, Nadembega C, Guenné S, Buñay J, Mahoukèdè Zohoncon T, Wendkuuni Djigma F, et al. Chemical composition, antioxidant and cytotoxic activities of *Hyptis suaveolens* (L.) Poit. essential oil on prostate and cervical cancers cells. Pak J Biol Sci. 2020;23(9):1184–92. doi: 10.3923/pjbs.2020.1184.1192 32981249

[17] da Silva ST, Coelho AD, de Assis RMA, de Carvalho AA, Bertolucci SKV, Pinto JEBP. Influence of light quality and some growth regulators in inducing the production of Podophyllotoxin, a bioactive compound against cancer, in adventitious roots formed in the leaves of *Hyptis suaveolens*. Ind Crops Prod. 2022;188:115710. doi: 10.1016/j.indcrop.2022.115710

[18] de Souza MCG, de Morais MB, da Andrade MS, de Vasconcelos MA, Sampaio SS, de Albuquerque CC. Mycorrhization and saline stress response in *Hyptis suaveolens*. Cienc Rural. 2020;50(6). doi: 10.1590/0103-8478cr20190533

[19] Grassi P, Nuñez MJ, Reyes TSU, Franz C. Chemical Variation in the Essential Oil Composition of *Hyptis suaveolens* (L.) Poit. (Lamiaceae). Nat Prod Commun. 2008;3(7). doi: 10.1177/1934578x0800300719

[20] Moola AK, Ayyadurai T, Balasubramani S, Vignesh R, Mohan PK, Sathish S, et al. Chemical composition and larvicidal activity against *Aedes aegypti* larvae from *Hyptis suaveolens* (L.) Poit essential oil. J Nat Pesticide Res. 2023;3:100018. doi: 10.1016/j.napere.2022.100018

[21] Salifou S, Houngnimassoun HMA, Dotche IO, Attindehou S, Salifou S. Larvicide activity of two chemotypes of *Hyptis suaveolens* (Lamiaceae) poit, 1806 and alphacypermethrin on larvae of *Rhipicepalus* (*Boophilus*) *microplus* (Can., 1887) (Acari: ixodidae). J Entomol Zool Stud. 2020;8:790–4.

[22] Sedano-Partida MD, dos Santos KP, Sala-Carvalho WR, Silva-Luz CL, Furlan CM. A review of the phytochemical profiling and biological activities of *Hyptis* Jacq.: a Brazilian native genus of Lamiaceae. Braz J Bot. 2020;43(1):213–28. doi: 10.1007/s40415-020-00582-y

[23] Abagli ZA, Alavo BCT. Potentialités insectifuges du gros baume, *Hyptis suaveolens* Poit. (Lamiaceae) : perspectives pour la lutte contre les moustiques en zones tropicales. JABs. 2020;149:15330–7. doi: 10.35759/jabs.149.7

[24] Htun NN, Sann S. Morphological identification of *Hyptis suaveolens* (L.) Poit. and its antimicrobial activity. Myanmar Korea Conf Res J. 2020;3:571–6.

[25] Ahoton LE, Alavo TBC, Ahomadegbe MA, Ahanhanzo C, Agbangla C. Domestication du gros baume (*Hyptis suaveolens* (L.) Poit.) : techniques de production et potentiels insectes ravageurs au sud du Bénin. Int J Biol Chem Sci. 2010;4(3). doi: 10.4314/ijbcs.v4i3.60463

[26] Mahajan V, Rather IA, Awasthi P, Anand R, Gairola S, Meena SR, et al. Development of chemical and EST-SSR markers for *Ocimum* genus. Industrial Crops and Products. 2015;63:65–70. doi: 10.1016/j.indcrop.2014.10.052

[27] Vieira R, Grayer R, Paton A, Simon J. Genetic diversity of *Ocimum gratissimum* L. based on volatile oil constituents, flavonoids and RAPD markers. Biochem Syst Ecol. 2001;29(3):287–304. doi: 10.1016/s0305-1978(00)00062-4 11152947

[28] Alves RP, Silva AVC, Almeida-Pereira CS, Costa TS, Alvares-Carvalho SV, Arrigoni-Blank M de F, et al. Genetic divergence in basil cultivars and hybrids. Hortic Bras. 2019;37(2):180–7. doi: 10.1590/s0102-053620190208

[29] Chowdhury T, Mandal A, Roy SC, De Sarker D. Diversity of the genus *Ocimum* (Lamiaceae) through morpho-molecular (RAPD) and chemical (GC-MS) analysis. J Genet Eng Biotechnol. 2017;15(1):275–86. doi: 10.1016/j.jgeb.2016.12.004 30647664 PMC6296643

[30] Afolayan AJ. Germination and growth features of seed of different sizes in *Hyptis suaveolens* (L.) Poit. Range Manag Agroforest. 1993;14:139–45.

[31] Gadidasu KK, Murthy EN, Nataraj P, Srinivas K, Babu PA, Raju VS. ISSR markers reveal genetic polymorphism in two morphological variants of *Hyptis suaveolens* invasive to India. Med Aromatic Plant Sci Biotechnol. 2011;5:166–163.

[32] Ji R, Yu X, Ren T, Chang Y, Li Z, Xia X, et al. Genetic diversity and population structure of *Caryopteris mongholica* revealed by reduced representation sequencing. BMC Plant Biol. 2022;22(1):297. doi: 10.1186/s12870-022-03681-y 35710341 PMC9205053

[33] Zheng Z, Sun Z, Fang Y, Qi F, Liu H, Miao L, et al. Genetic Diversity, Population Structure, and Botanical Variety of 320 Global Peanut Accessions Revealed Through Tunable Genotyping-by-Sequencing. Sci Rep. 2018;8(1):14500. doi: 10.1038/s41598-018-32800-9 30266974 PMC6162295

[34] Arbon PM, Silva CNS, Jones DB, Jaccoud D, Gervis M, Jerry DR, et al. Development and validation of a SNP-based genotyping tool for pedigree establishment in Australian greenlip abalone *Haliotis laevigata* Donovan, 1808. Aquacult Rep. 2021;20:100746. doi: 10.1016/j.aqrep.2021.100746

[35] Ariani A, Berny Mier y Teran JC, Gepts P. Genome-wide identification of SNPs and copy number variation in common bean (*Phaseolus vulgaris* L.) using genotyping-by-sequencing (GBS). Mol Breeding. 2016;36(7). doi: 10.1007/s11032-016-0512-9

[36] van Deventer R, Rhode C, Marx M, Roodt-Wilding R. The development of genome-wide single nucleotide polymorphisms in blue wildebeest using the DArTseq platform. Genomics. 2020;112(5):3455–64. doi: 10.1016/j.ygeno.2020.04.032 32574831

[37] Ali Koura A, Wireko Kena A, Annor B, Adejumobi II, Maina F, Maazou A-RS, et al. DArTseq-based genome-wide SNP markers reveal limited genetic diversity and highly structured population in assembled West African cowpea germplasm. Sci Afr. 2024;23:e02065. doi: 10.1016/j.sciaf.2024.e02065

[38] Gossa AG, Tesfaye B, Degu HD. Genetic diversity and population structure of Ethiopian basil (*Ocimum basilicum* L.) accessions using DArTseq markers. Ecol Gene Genom. 2024;31:100241. doi: 10.1016/j.egg.2024.100241

[39] Fufa TW, Abtew WG, Amadi CO, Oselebe HO. DArTSeq SNP-based genetic diversity and population structure studies among taro [(*Colocasia esculenta* (L.) Schott] accessions sourced from Nigeria and Vanuatu. PLoS One. 2022;17(11):e0269302. doi: 10.1371/journal.pone.0269302 36355796 PMC9648780

[40] Tchokponhoué DA, Achigan-Dako EG, Sognigbé N, Nyadanu D, Hale I, Odindo AO, et al. Genome-wide diversity analysis suggests divergence among Upper Guinea and the Dahomey Gap populations of the Sisrè berry (Syn: miracle fruit) plant (*Synsepalum dulcificum* [Schumach. & Thonn.] Daniell) in West Africa. Plant Genome. 2023;16(1):e20299. doi: 10.1002/tpg2.20299 36661287 PMC12807384

[41] Bocianowski J, Tomkowiak A, Bocianowska M, Sobiech A. The use of dartseq technology to identify markers related to the heterosis effects in selected traits in Maize. Curr Issues Mol Biol. 2023;45(4):2644–60. doi: 10.3390/cimb45040173 37185697 PMC10136425

[42] Gelaw YM, Eleblu JSY, Ofori K, Fenta BA, Mukankusi C, Emam EA, et al. High-density DArTSeq SNP markers revealed wide genetic diversity and structured population in common bean (*Phaseolus vulgaris* L.) germplasm in Ethiopia. Mol Biol Rep. 2023;50(8):6739–51. doi: 10.1007/s11033-023-08498-y 37389701 PMC10374692

[43] Capo-Chichi DBE, Tchokponhoué DA, Sogbohossou DEO, Achigan-Dako EG. Narrow genetic diversity in germplasm from the Guinean and Sudano-Guinean zones in Benin indicates the need to broaden the genetic base of sweet fig banana (*Musa acuminata* cv Sotoumon). PLoS One. 2023;18(11):e0294315. doi: 10.1371/journal.pone.0294315 37972084 PMC10653437

[44] Akohoue F, Achigan-Dako EG, Sneller C, Van Deynze A, Sibiya J. Genetic diversity, SNP-trait associations and genomic selection accuracy in a west African collection of Kersting’s groundnut [*Macrotyloma geocarpum*(Harms) Maréchal & Baudet]. PLoS One. 2020;15(6):e0234769. doi: 10.1371/journal.pone.0234769 32603370 PMC7326195

[45] Akoègninou A, van der Burg WJ, van der Maesen LJG. Flore analytique du Bénin. Backhuys Publishers; 2006. Available from: https://research.wur.nl/en/publications/flore-analytique-du-b%C3%A9nin

[46] Kilian A, Wenzl P, Huttner E, Carling J, Xia L, Blois H, et al. Diversity arrays technology: a generic genome profiling technology on open platforms. Data production and analysis in population genomics: Methods and protocols. 2012. pp. 67–89. 10.1007/978-1-61779-870-2_5 22665276

[47] Sansaloni C, Petroli C, Jaccoud D, Carling J, Detering F, Grattapaglia D, et al. Diversity Arrays Technology (DArT) and next-generation sequencing combined: genome-wide, high throughput, highly informative genotyping for molecular breeding of Eucalyptus. BMC Proc. 2011;5(S7). doi: 10.1186/1753-6561-5-s7-p54

[48] Gruber B, Unmack PJ, Berry OF, Georges A. dartr: An r package to facilitate analysis of SNP data generated from reduced representation genome sequencing. Mol Ecol Resour. 2018;18(3):691–9. doi: 10.1111/1755-0998.12745 29266847

[49] Pereira VM, Filho JAF, Leão AP, Vargas LHG, de Farias MP, de Rios SA, et al. American oil palm from Brazil: Genetic diversity, population structure, and core collection. Crop Sci. 2020;60(6):3212–27. doi: 10.1002/csc2.20276

[50] Kamvar ZN, Tabima JF, Grünwald NJ. Poppr: an R package for genetic analysis of populations with clonal, partially clonal, and/or sexual reproduction. PeerJ. 2014;2:e281. doi: 10.7717/peerj.281 24688859 PMC3961149

[51] Weir BS, Cockerham CC. Estimating F-statistics for the analysis of population structure. Evolution. 1984:1358–70. 28563791 10.1111/j.1558-5646.1984.tb05657.x

[52] Pritchard JK, Stephens M, Donnelly P. Inference of population structure using multilocus genotype data. Genetics. 2000;155(2):945–59. doi: 10.1093/genetics/155.2.945 10835412 PMC1461096

[53] Earl DA, vonHoldt BM. STRUCTURE HARVESTER: a website and program for visualizing STRUCTURE output and implementing the Evanno method. Conserv Genet Resour. 2011;4(2):359–61. doi: 10.1007/s12686-011-9548-7

[54] Evanno G, Regnaut S, Goudet J. Detecting the number of clusters of individuals using the software STRUCTURE: a simulation study. Mol Ecol. 2005;14(8):2611–20. doi: 10.1111/j.1365-294X.2005.02553.x 15969739

[55] Ketema S, Tesfaye B, Keneni G, Amsalu Fenta B, Assefa E, Greliche N, et al. DArTSeq SNP-based markers revealed high genetic diversity and structured population in Ethiopian cowpea [*Vigna unguiculata* (L.) Walp] germplasms. PLoS One. 2020;15(10):e0239122. doi: 10.1371/journal.pone.0239122 33031381 PMC7544073

[56] Kumar S, Stecher G, Li M, Knyaz C, Tamura K. MEGA X: molecular evolutionary genetics analysis across computing platforms. Mol Biol Evol. 2018;35(6):1547–9. doi: 10.1093/molbev/msy096 29722887 PMC5967553

[57] Letunic I, Bork P. Interactive Tree Of Life (iTOL) v5: an online tool for phylogenetic tree display and annotation. Nucleic Acids Res. 2021;49(W1):W293–6. doi: 10.1093/nar/gkab301 33885785 PMC8265157

[58] Gao Y, Yin S, Wu L, Dai D, Wang H, Liu C, et al. Genetic diversity and structure of wild and cultivated *Amorphophallus paeoniifolius* populations in southwestern China as revealed by RAD-seq. Sci Rep. 2017;7(1):14183. doi: 10.1038/s41598-017-14738-6 29079830 PMC5660214

[59] Feitosa-Alcantara RB, da Muniz AVCS, Blank AF, Almeida-Pereira CS, Carvalho SVÁ, Arrigoni-Blank M de F. Analysis of genetic diversity of *Hyptis pectinata* (L.) Poit. plants using ISSR markers. 2017;16. 10.4238/gmr16039603 28829892

[60] Kyrkjeeide MO, Westergaard KB, Kleven O, Evju M, Endrestøl A, Brandrud MK, et al. Conserving on the edge: genetic variation and structure in northern populations of the endangered plant *Dracocephalum ruyschiana* L. (Lamiaceae). Conserv Genet. 2020;21(4):707–18. doi: 10.1007/s10592-020-01281-7

[61] Zhang C, Jia C, Liu X, Zhao H, Hou L, Li M, et al. Genetic Diversity Study on Geographical Populations of the Multipurpose Species *Elsholtzia stauntonii* Using Transferable Microsatellite Markers. Front Plant Sci. 2022;13:903674. doi: 10.3389/fpls.2022.903674 35646027 PMC9134938

[62] Aluri JSR, Reddi C. Pollination ecology of *Hyptis suaveolens* (Lamiaceae). Proc Natl Acad Sci. 1989;B55:411–6.

[63] Söderquist L, Karrenberg S, Sletvold N. Genetic differentiation and diversity do not explain variation in heterosis or inbreeding depression: empirical evidence from a long-lived iteroparous plant. Conserv Genet. 2024;25(6):1303–12. doi: 10.1007/s10592-024-01641-7

[64] Luo Z, Brock J, Dyer JM, Kutchan T, Schachtman D, Augustin M, et al. Genetic Diversity and Population Structure of a *Camelina sativa* Spring Panel. Front Plant Sci. 2019;10:184. doi: 10.3389/fpls.2019.00184 30842785 PMC6391347

[65] Wright S. Evolution and the Genetics of Populations, Volume 4: Variability Within and Among Natural Populations. Chicago, IL: University of Chicago Press; 1984. Available from: https://press.uchicago.edu/ucp/books/book/chicago/E/bo3642015.html

[66] Dostálek T, Münzbergová Z, Plačková I. Genetic diversity and its effect on fitness in an endangered plant species, *Dracocephalum austriacum* L. Conserv Genet. 2009;11(3):773–83. doi: 10.1007/s10592-009-9879-z

[67] Whitlock MC, McCauley DE. Indirect measures of gene flow and migration: FST not equal to 1/(4Nm + 1). Heredity (Edinb). 1999;82 ( Pt 2):117–25. doi: 10.1038/sj.hdy.6884960 10098262

[68] Yao X, Ye Q, Kang M, Huang H. Microsatellite analysis reveals interpopulation differentiation and gene flow in the endangered tree *Changiostyrax dolichocarpa* (Styracaceae) with fragmented distribution in central China. New Phytol. 2007;176(2):472–80. doi: 10.1111/j.1469-8137.2007.02175.x 17888124

[69] Coelho AD, de Assis RMA, Leite JJF, Rocha JPM, Bertolucci SKV, Pinto JEBP. Podophyllotoxin bioaccumulation with biotic and abiotic elicitors on root culture of *Hyptis suaveolens* (L.) POIT. In Vitro CellDevBiol-Plant. 2025;61(1):51–66. doi: 10.1007/s11627-024-10482-5

[70] Coelho AD, Mendonça SC, de Assis RMA, Rocha JPM, Shukla MR, Saxena PK, et al. Impact of phloroglucinol and indoleamines on growth and accumulation of podophyllotoxin in the roots of *Hyptis suaveolens* (L.) Poit. Plant Cell Tiss Organ Cult. 2024;158(3). doi: 10.1007/s11240-024-02845-y

[71] Francini A, Pintado M, Manganaris GA, Ferrante A. Editorial: Bioactive Compounds Biosynthesis and Metabolism in Fruit and Vegetables. Front Plant Sci. 2020;11:129. doi: 10.3389/fpls.2020.00129 32140170 PMC7042200

[72] Shahhat IM, Elsheikh SYS. Accumulation of secondary metabolites in the family Lamiaceae as influenced by foliar micronutrients. Boletín Latinoamericano y del Caribe de Plantas Medicinales y Aromáticas. 2024;23(6):823–38. doi: 10.37360/blacpma.24.23.6.53

[73] de Melo ATO, Coelho ASG, Pereira MF, Blanco AJV, Franceschinelli EV. High genetic diversity and strong spatial genetic structure in *Cabralea canjerana* (Vell.) Mart. (Meliaceae): implications to Brazilian Atlantic Forest tree conservation. Natureza Conservação. 2014;12(2):129–33. doi: 10.1016/j.ncon.2014.08.001

[74] Pyne RM, Honig JA, Vaiciunas J, Wyenandt CA, Simon JE. Population structure, genetic diversity and downy mildew resistance among *Ocimum* species germplasm. BMC Plant Biol. 2018;18(1):69. doi: 10.1186/s12870-018-1284-7 29685108 PMC5914031

[75] Loveless MD, Hamrick JL. Ecological determinants of genetic structure in plant populations. Annu Rev Ecol Syst. 1984;15(1):65–95. doi: 10.1146/annurev.es.15.110184.000433

[76] Sedano-Partida MD. Chemical and biological potential of Hyptis Jacq. (Lamiaceae). Universidade de São Paulo; 2018.

