# Supplementary material for: SNP markers revealed the genetic diversity and population structure of Mesosphaerum suaveolens (L.) Kuntze Syn. Hyptis suaveolens (L.) Poit accessions collected in Benin
Source: PLoS One. 2025 Sep 4;20(9):e0331702. doi: 10.1371/journal.pone.0331702 (PMC12410747; doi:10.1371/journal.pone.0331702)
Supplement: S1 Table — This is a word file presenting the coordinates of each accession sampled. (DOCX) [file pone.0331702.s001.docx]

|  | S1 Table. Coordinates for each accession from the three phytogeographical regions of Benin | | | | | |
| --- | --- | --- | --- | --- | --- | --- |
| Accessions | Voucher number | Phytogeographical regions | Departments | Communes | Latitude | Longitude |
| 1 | GBS-HYS-0001 | Sudano-Guinean | Borgou | Parakou | 9.350278 | 2.619167 |
| 4 | GBS-HYS-0002 | Sudano-Guinean | Borgou | Parakou | 9.345 | 2.559722 |
| 8 | GBS-HYS-0003 | Sudano-Guinean | Borgou | Parakou | 9.368611 | 2.6125 |
| 9 | GBS-HYS-0004 | Sudano-Guinean | Borgou | Parakou | 9.368611 | 2.6125 |
| 10 | GBS-HYS-0005 | Sudano-Guinean | Borgou | Parakou | 9.368611 | 2.6125 |
| 11 | GBS-HYS-0006 | Sudano-Guinean | Borgou | Parakou | 9.350833 | 2.6125 |
| 13 | GBS-HYS-0007 | Sudano-Guinean | Borgou | Parakou | 9.366667 | 2.619444 |
| 14 | GBS-HYS-0008 | Sudano-Guinean | Borgou | Parakou | 9.366667 | 2.566667 |
| 15 | GBS-HYS-0009 | Sudano-Guinean | Borgou | Parakou | 9.366667 | 2.622222 |
| 17 | GBS-HYS-0010 | Sudano-Guinean | Borgou | Parakou | 9.350278 | 2.619167 |
| 18 | GBS-HYS-0011 | Sudano-Guinean | Borgou | Parakou | 9.350278 | 2.619167 |
| 19 | GBS-HYS-0012 | Sudano-Guinean | Borgou | Parakou | 9.350278 | 2.619167 |
| 24 | GBS-HYS-0013 | Sudano-Guinean | Borgou | Ndali | 9.854722 | 2.708611 |
| 26 | GBS-HYS-0014 | Sudano-Guinean | Borgou | Ndali | 9.854722 | 2.541944 |
| 28 | GBS-HYS-0015 | Sudano-Guinean | Borgou | Ndali | 9.751667 | 2.540556 |
| 29 | GBS-HYS-0016 | Sudano-Guinean | Borgou | Ndali | 9.731944 | 2.507222 |
| 30 | GBS-HYS-0017 | Sudano-Guinean | Borgou | Ndali | 9.731944 | 2.507222 |
| 31 | GBS-HYS-0018 | Sudano-Guinean | Borgou | Ndali | 9.785278 | 2.439444 |
| 33 | GBS-HYS-0019 | Sudano-Guinean | Borgou | Ndali | 9.785278 | 2.489444 |
| 34 | GBS-HYS-0020 | Sudanian | Donga | Copargo | 9.784167 | 1.578333 |
| 35 | GBS-HYS-0021 | Sudanian | Donga | Copargo | 9.800833 | 1.578333 |
| 36 | GBS-HYS-0022 | Sudanian | Donga | Copargo | 9.784167 | 1.678333 |
| 38 | GBS-HYS-0023 | Sudanian | Donga | Copargo | 9.784167 | 1.578333 |
| 40 | GBS-HYS-0024 | Sudanian | Donga | Copargo | 9.854167 | 1.538611 |
| 41 | GBS-HYS-0025 | Sudanian | Donga | Copargo | 9.854167 | 1.538611 |
| 43 | GBS-HYS-0026 | Sudanian | Donga | Copargo | 9.822778 | 1.555833 |
| 44 | GBS-HYS-0027 | Sudanian | Donga | Copargo | 9.822778 | 1.555833 |
| 45 | GBS-HYS-0028 | Sudano-Guinean | Donga | Djougou | 9.656111 | 1.719722 |
| 47 | GBS-HYS-0029 | Sudano-Guinean | Donga | Djougou | 9.656111 | 1.719722 |
| 52 | GBS-HYS-0030 | Sudanian | Donga | Djougou | 9.7075 | 1.679444 |
| 53 | GBS-HYS-0031 | Sudanian | Donga | Djougou | 9.7075 | 1.679444 |
| 55 | GBS-HYS-0032 | Sudano-Guinean | Donga | Djougou | 9.668889 | 1.697778 |
| 57 | GBS-HYS-0033 | Sudanian | Donga | Djougou | 9.722778 | 1.776667 |
| 61 | GBS-HYS-0034 | Sudanian | Atacora | Natitingou | 10.328056 | 1.38 |
| 66 | GBS-HYS-0035 | Sudanian | Atacora | Natitingou | 10.300833 | 1.386667 |
| 68 | GBS-HYS-0036 | Sudanian | Atacora | Natitingou | 10.300833 | 1.386667 |
| 69 | GBS-HYS-0037 | Sudano-Guinean | Borgou | Ndali | 9.741667 | 2.440556 |
| 70 | GBS-HYS-0038 | Sudano-Guinean | Borgou | Ndali | 9.741667 | 2.440556 |
| 71 | GBS-HYS-0039 | Sudano-Guinean | Borgou | Ndali | 9.741667 | 2.440556 |
| 74 | GBS-HYS-0040 | Sudanian | Atacora | Boukombé | 10.145278 | 1.162222 |
| 77 | GBS-HYS-0041 | Sudanian | Atacora | Boukombé | 10.239444 | 1.015833 |
| 79 | GBS-HYS-0042 | Sudanian | Atacora | Boukombé | 10.255833 | 0.9925 |
| 82 | GBS-HYS-0043 | Sudanian | Atacora | Boukombé | 10.195 | 1.085 |
| 84 | GBS-HYS-0044 | Sudanian | Atacora | Boukombé | 10.18 | 1.201944 |
| 86 | GBS-HYS-0045 | Sudanian | Alibori | Gogounou | 10.733333 | 2.966667 |
| 87 | GBS-HYS-0046 | Sudanian | Alibori | Gogounou | 10.733333 | 2.966667 |
| 88 | GBS-HYS-0047 | Sudanian | Alibori | Gogounou | 10.733333 | 2.966667 |
| 89 | GBS-HYS-0048 | Sudanian | Alibori | Gogounou | 10.716667 | 2.966667 |
| 90 | GBS-HYS-0049 | Sudanian | Alibori | Gogounou | 10.733333 | 2.95 |
| 91 | GBS-HYS-0050 | Sudanian | Alibori | Gogounou | 10.7 | 2.966667 |
| 92 | GBS-HYS-0051 | Sudanian | Alibori | Gogounou | 10.814722 | 2.716389 |
| 98 | GBS-HYS-0052 | Sudanian | Alibori | Gogounou | 10.733333 | 2.966667 |
| 102 | GBS-HYS-0053 | Sudanian | Alibori | Gogounou | 10.843056 | 2.828333 |
| 103 | GBS-HYS-0054 | Sudanian | Alibori | Kandi | 11.128611 | 2.936944 |
| 104 | GBS-HYS-0055 | Sudanian | Alibori | Kandi | 11.128611 | 2.936944 |
| 105 | GBS-HYS-0056 | Sudanian | Alibori | Kandi | 11.128611 | 2.936944 |
| 106 | GBS-HYS-0057 | Sudanian | Alibori | Kandi | 11.128611 | 2.936944 |
| 109 | GBS-HYS-0058 | Sudanian | Alibori | Kandi | 11.133056 | 2.935278 |
| 110 | GBS-HYS-0059 | Sudanian | Alibori | Kandi | 11.133056 | 2.935278 |
| 111 | GBS-HYS-0060 | Sudanian | Alibori | Kandi | 11.133056 | 2.935278 |
| 114 | GBS-HYS-0061 | Sudanian | Alibori | Kandi | 11.133333 | 2.933333 |
| 118 | GBS-HYS-0062 | Sudano-Guinean | Collines | Glazoué | 8.586944 | 2.343889 |
| 119 | GBS-HYS-0063 | Sudano-Guinean | Collines | Glazoué | 8.603611 | 2.377222 |
| 124 | GBS-HYS-0064 | Sudano-Guinean | Collines | Panhouian | 7.677778 | 2.226944 |
| 126 | GBS-HYS-0065 | Sudano-Guinean | Collines | Panhouian | 7.677778 | 2.226944 |
| 127 | GBS-HYS-0066 | Sudano-Guinean | Collines | Panhouian | 7.677778 | 2.226944 |
| 129 | GBS-HYS-0067 | Sudano-Guinean | Collines | Panhouian | 7.677778 | 2.226944 |
| 130 | GBS-HYS-0068 | Sudano-Guinean | Collines | Panhouian | 7.677778 | 2.226944 |
| 132 | GBS-HYS-0069 | Sudano-Guinean | Collines | Panhouian | 7.677778 | 2.226944 |
| 134 | GBS-HYS-0070 | Sudano-Guinean | Collines | Savè | 8.243889 | 2.685556 |
| 135 | GBS-HYS-0071 | Sudano-Guinean | Collines | Savè | 8.210556 | 2.685556 |
| 137 | GBS-HYS-0072 | Sudano-Guinean | Collines | Savè | 8.227222 | 2.685556 |
| 138 | GBS-HYS-0073 | Sudano-Guinean | Collines | Savè | 8.243889 | 2.685556 |
| 139 | GBS-HYS-0074 | Sudano-Guinean | Collines | Savè | 8.243889 | 2.685556 |
| 141 | GBS-HYS-0075 | Sudano-Guinean | Collines | Savè | 8.243889 | 2.685556 |
| 143 | GBS-HYS-0076 | Sudano-Guinean | Collines | Dassa | 7.786389 | 2.191389 |
| 144 | GBS-HYS-0077 | Sudano-Guinean | Collines | Dassa | 7.786389 | 2.191389 |
| 147 | GBS-HYS-0078 | Sudano-Guinean | Collines | Dassa | 7.786389 | 2.191389 |
| 151 | GBS-HYS-0079 | Guineo-Congolian | Ouémé | Bonou | 6.819444 | 2.471944 |
| 152 | GBS-HYS-0080 | Guineo-Congolian | Ouémé | Bonou | 6.819444 | 2.471944 |
| 153 | GBS-HYS-0081 | Guineo-Congolian | Ouémé | Bonou | 6.819444 | 2.471944 |
| 155 | GBS-HYS-0082 | Guineo-Congolian | Ouémé | Bonou | 6.819444 | 2.471944 |
| 157 | GBS-HYS-0083 | Guineo-Congolian | Ouémé | Sèmè-kpodji | 6.378056 | 2.584167 |
| 158 | GBS-HYS-0084 | Guineo-Congolian | Ouémé | Sèmè-kpodji | 6.378056 | 2.584167 |
| 159 | GBS-HYS-0085 | Guineo-Congolian | Ouémé | Sèmè-kpodji | 6.378056 | 2.584167 |
| 164 | GBS-HYS-0086 | Guineo-Congolian | Ouémé | Dangbo | 6.630556 | 2.533611 |
| 165 | GBS-HYS-0087 | Guineo-Congolian | Ouémé | Dangbo | 6.623333 | 2.537222 |
| 166 | GBS-HYS-0088 | Guineo-Congolian | Ouémé | Adjohoun | 6.733333 | 2.483611 |
| 167 | GBS-HYS-0089 | Guineo-Congolian | Ouémé | Adjohoun | 6.662778 | 2.5025 |
| 168 | GBS-HYS-0090 | Guineo-Congolian | Ouémé | Adjohoun | 6.736667 | 2.481944 |
| 170 | GBS-HYS-0091 | Guineo-Congolian | Ouémé | Adjohoun | 6.371944 | 2.394167 |
| 171 | GBS-HYS-0092 | Guineo-Congolian | Ouémé | Adjohoun | 6.371944 | 2.394167 |
| 172 | GBS-HYS-0093 | Guineo-Congolian | Ouémé | Adjohoun | 6.662778 | 2.5025 |
| 173 | GBS-HYS-0094 | Guineo-Congolian | Ouémé | Adjohoun | 6.736667 | 2.481944 |
| 177 | GBS-HYS-0095 | Guineo-Congolian | Ouémé | Adjohoun | 6.736667 | 2.481944 |
| 178 | GBS-HYS-0096 | Guineo-Congolian | Mono | Comè | 6.436944 | 1.844167 |
| 180 | GBS-HYS-0097 | Guineo-Congolian | Mono | Comè | 6.417222 | 1.874722 |
| 183 | GBS-HYS-0098 | Guineo-Congolian | Mono | Comè | 6.465833 | 1.920556 |
| 184 | GBS-HYS-0099 | Guineo-Congolian | Mono | Comè | 6.536667 | 1.861944 |
| 185 | GBS-HYS-0100 | Guineo-Congolian | Mono | Comè | 6.46 | 1.9225 |
| 186 | GBS-HYS-0101 | Guineo-Congolian | Mono | Comè | 6.465833 | 1.920556 |
| 187 | GBS-HYS-0102 | Guineo-Congolian | Mono | Comè | 6.465833 | 1.920556 |
| 189 | GBS-HYS-0103 | Guineo-Congolian | Mono | Grand-popo | 6.388611 | 1.821111 |
| 191 | GBS-HYS-0104 | Guineo-Congolian | Mono | Grand-popo | 6.375556 | 1.8675 |
| 194 | GBS-HYS-0105 | Guineo-Congolian | Mono | Grand-popo | 6.388611 | 1.821111 |
| 195 | GBS-HYS-0106 | Guineo-Congolian | Mono | Grand-popo | 6.388611 | 1.821111 |
| 198 | GBS-HYS-0107 | Guineo-Congolian | Mono | Bopa | 6.514722 | 1.950833 |
| 199 | GBS-HYS-0108 | Guineo-Congolian | Mono | Bopa | 6.522778 | 1.969167 |
| 200 | GBS-HYS-0109 | Guineo-Congolian | Mono | Comè | 6.436944 | 1.844167 |
| 202 | GBS-HYS-0110 | Guineo-Congolian | Mono | Houéyogbé | 6.521111 | 1.820278 |
| 203 | GBS-HYS-0111 | Guineo-Congolian | Atlantique | Ouidah | 2.368056 | 2.129722 |
| 205 | GBS-HYS-0112 | Guineo-Congolian | Atlantique | Ouidah | 2.368056 | 2.129722 |
| 209 | GBS-HYS-0113 | Guineo-Congolian | Atlantique | Ouidah | 2.368056 | 2.129722 |
| 211 | GBS-HYS-0114 | Guineo-Congolian | Atlantique | Ouidah | 6.357778 | 2.077222 |
| 212 | GBS-HYS-0115 | Guineo-Congolian | Atlantique | kpomasse | 6.363889 | 2.078889 |
| 213 | GBS-HYS-0116 | Guineo-Congolian | Atlantique | Ouidah | 2.368056 | 2.129722 |
| 214 | GBS-HYS-0117 | Guineo-Congolian | Atlantique | Ouidah | 2.368056 | 2.129722 |
| 218 | GBS-HYS-0118 | Guineo-Congolian | Atlantique | Ouidah | 2.368056 | 2.129722 |
| 219 | GBS-HYS-0119 | Guineo-Congolian | Atlantique | Ouidah | 6.331389 | 2.065 |
| 220 | GBS-HYS-0120 | Guineo-Congolian | Atlantique | Abomey-calavi | 6.467222 | 2.318056 |
| 222 | GBS-HYS-0121 | Guineo-Congolian | Atlantique | Abomey-calavi | 6.467222 | 2.318056 |
| 224 | GBS-HYS-0122 | Guineo-Congolian | Atlantique | Abomey-calavi | 6.467222 | 2.318056 |
| 225 | GBS-HYS-0123 | Guineo-Congolian | Atlantique | Abomey-calavi | 6.467222 | 2.318056 |
| 226 | GBS-HYS-0124 | Guineo-Congolian | Atlantique | Abomey-calavi | 6.467222 | 2.318056 |
| 227 | GBS-HYS-0125 | Guineo-Congolian | Atlantique | Abomey-calavi | 6.435556 | 2.252778 |
| 228 | GBS-HYS-0126 | Guineo-Congolian | Atlantique | Abomey-calavi | 6.467222 | 2.318056 |
| 230 | GBS-HYS-0127 | Guineo-Congolian | Couffo | Djakotomey | 6.921667 | 1.700278 |
| 231 | GBS-HYS-0128 | Guineo-Congolian | Couffo | Djakotomey | 6.921667 | 1.700278 |
| 233 | GBS-HYS-0129 | Guineo-Congolian | Couffo | Djakotomey | 6.921667 | 1.700278 |
| 234 | GBS-HYS-0130 | Guineo-Congolian | Couffo | Djakotomey | 6.921667 | 1.700278 |
| 235 | GBS-HYS-0131 | Guineo-Congolian | Couffo | Djakotomey | 6.921667 | 1.700278 |
| 237 | GBS-HYS-0132 | Guineo-Congolian | Couffo | Djakotomey | 6.921667 | 1.700278 |
| 239 | GBS-HYS-0133 | Guineo-Congolian | Couffo | Djakotomey | 6.921667 | 1.700278 |
| 240 | GBS-HYS-0134 | Guineo-Congolian | Couffo | Azovê | 6.950833 | 1.694722 |
| 242 | GBS-HYS-0135 | Guineo-Congolian | Couffo | Azovê | 6.950833 | 1.694722 |
| 243 | GBS-HYS-0136 | Guineo-Congolian | Couffo | Azovê | 6.950833 | 1.694722 |
| 245 | GBS-HYS-0137 | Guineo-Congolian | Couffo | Dogbo | 6.790833 | 1.773056 |
| 250 | GBS-HYS-0138 | Guineo-Congolian | Couffo | Aplahoué | 6.941389 | 1.700278 |
| 251 | GBS-HYS-0139 | Guineo-Congolian | Couffo | Aplahoué | 6.941389 | 1.700278 |
| 254 | GBS-HYS-0140 | Guineo-Congolian | Plteau | sakété | 6.419167 | 2.335556 |
| 255 | GBS-HYS-0141 | Guineo-Congolian | Plteau | sakété | 6.419167 | 2.335556 |
| 256 | GBS-HYS-0142 | Guineo-Congolian | Plteau | sakété | 6.419167 | 2.335556 |
| 260 | GBS-HYS-0143 | Guineo-Congolian | Plteau | sakété | 6.419167 | 2.335556 |
| 262 | GBS-HYS-0144 | Guineo-Congolian | Plteau | sakété | 6.419167 | 2.335556 |
| 264 | GBS-HYS-0145 | Guineo-Congolian | Plteau | sakété | 6.419167 | 2.335556 |
| 265 | GBS-HYS-0146 | Guineo-Congolian | Plteau | Pobè | 6.954167 | 2.335556 |
| 266 | GBS-HYS-0147 | Guineo-Congolian | Plteau | Pobè | 6.954167 | 2.335556 |
| 268 | GBS-HYS-0148 | Guineo-Congolian | Plteau | Akpro-misserété | 6.544444 | 2.594444 |
| 269 | GBS-HYS-0149 | Guineo-Congolian | Zou | Za-kpota | 7.200278 | 2.141667 |
| 270 | GBS-HYS-0150 | Guineo-Congolian | Zou | Za-kpota | 7.200278 | 2.141667 |
| 271 | GBS-HYS-0151 | Guineo-Congolian | Zou | Za-kpota | 7.200278 | 2.141667 |
| 275 | GBS-HYS-0152 | Guineo-Congolian | Zou | Za-kpota | 7.200278 | 2.141667 |
| 276 | GBS-HYS-0153 | Guineo-Congolian | Zou | Za-kpota | 7.200278 | 2.141667 |
| 277 | GBS-HYS-0154 | Guineo-Congolian | Zou | Za-kpota | 7.200278 | 2.141667 |
| 279 | GBS-HYS-0155 | Guineo-Congolian | Zou | Za-kpota | 7.200278 | 2.141667 |
| 282 | GBS-HYS-0156 | Guineo-Congolian | Zou | Za-kpota | 7.200278 | 2.141667 |
| 283 | GBS-HYS-0157 | Guineo-Congolian | Zou | Zogbodomey | 7.075 | 2.109167 |
| 285 | GBS-HYS-0158 | Guineo-Congolian | Zou | Zogbodomey | 7.075 | 2.109167 |
| 286 | GBS-HYS-0159 | Guineo-Congolian | Zou | Zogbodomey | 7.075 | 2.109167 |
| 289 | GBS-HYS-0160 | Guineo-Congolian | Zou | Zogbodomey | 7.075 | 2.109167 |
| 290 | GBS-HYS-0161 | Guineo-Congolian | Zou | Zogbodomey | 7.075 | 2.109167 |
| 291 | GBS-HYS-0162 | Guineo-Congolian | Zou | Zogbodomey | 7.075 | 2.109167 |
| 292 | GBS-HYS-0163 | Guineo-Congolian | Zou | Bohicon | 7.259167 | 2.105556 |
| 293 | GBS-HYS-0164 | Guineo-Congolian | Zou | Bohicon | 7.255 | 2.099444 |
| 295 | GBS-HYS-0165 | Guineo-Congolian | Zou | Bohicon | 7.291667 | 2.091944 |
| 296 | GBS-HYS-0166 | Guineo-Congolian | Zou | Bohicon | 7.291667 | 2.091944 |
| 297 | GBS-HYS-0167 | Guineo-Congolian | Zou | Bohicon | 7.3175 | 2.088889 |
| 298 | GBS-HYS-0168 | Guineo-Congolian | Zou | Zakpota | 7.200278 | 2.141667 |
| 299 | GBS-HYS-0169 | Guineo-Congolian | Littoral | Cotonou | 6.351111 | 2.352778 |
| 300 | GBS-HYS-0170 | Guineo-Congolian | Littoral | Cotonou | 6.351111 | 2.352778 |
| 301 | GBS-HYS-0171 | Guineo-Congolian | Couffo | Djakotomey | 6.921667 | 1.712778 |
| 303 | GBS-HYS-0172 | Sudano-Guinean | Collines | Kpanhouignan | 7.711111 | 2.226944 |
| 304 | GBS-HYS-0173 | Sudano-Guinean | Collines | Kpanhouignan | 7.677778 | 2.226944 |
| 307 | GBS-HYS-0174 | Sudano-Guinean | Collines | Glazoué | 8.603611 | 2.377222 |
| 309 | GBS-HYS-0175 | Sudano-Guinean | Collines | Kpanhouignan | 7.761111 | 2.226944 |
