# Supplementary material for: SNP markers revealed the genetic diversity and population structure of Mesosphaerum suaveolens (L.) Kuntze Syn. Hyptis suaveolens (L.) Poit accessions collected in Benin
Source: PLoS One. 2025 Sep 4;20(9):e0331702. doi: 10.1371/journal.pone.0331702 (PMC12410747; doi:10.1371/journal.pone.0331702)
Supplement: S2 Table — Admixt = Admixed individuals, C1 = Individuals of the group 1 and C2 = Individuals of the group 2. This is a word file presenting the accessions grouping. (DOCX) [file pone.0331702.s002.docx]

S2 Table. *Mesosphaerum suaveolens* accessions grouping based of the membership value (Q). Admixt = Admixed individuals, C1 = Individuals of the group 1 and C2 = Individuals of the group 2.

|  |  |  |  |  |
| --- | --- | --- | --- | --- |
| **Accessions** | **Voucher number** | **Membership coefficient** | | **Final class** |
|  |  | **Group 1**  **(C1)** | **Group 2 (C2)** |  |
| 1 | GBS-HYS-0001 | 0.226 | 0.774 | C2 |
| 4 | GBS-HYS-0002 | 0.542 | 0.458 | Admixt |
| 8 | GBS-HYS-0003 | 0.914 | 0.086 | C2 |
| 9 | GBS-HYS-0004 | 0.129 | 0.871 | C2 |
| 10 | GBS-HYS-0005 | 0.104 | 0.896 | C2 |
| 11 | GBS-HYS-0006 | 0.602 | 0.398 | Admixt |
| 13 | GBS-HYS-0007 | 0.137 | 0.863 | C2 |
| 14 | GBS-HYS-0008 | 0.118 | 0.882 | C2 |
| 15 | GBS-HYS-0009 | 0.129 | 0.871 | C2 |
| 17 | GBS-HYS-0010 | 0.037 | 0.963 | C2 |
| 18 | GBS-HYS-0011 | 0.18 | 0.82 | C2 |
| 19 | GBS-HYS-0012 | 0.007 | 0.993 | C2 |
| 24 | GBS-HYS-0013 | 0.478 | 0.522 | Admixt |
| 26 | GBS-HYS-0014 | 0.411 | 0.589 | Admixt |
| 28 | GBS-HYS-0015 | 0.599 | 0.401 | Admixt |
| 29 | GBS-HYS-0016 | 0.08 | 0.92 | C2 |
| 30 | GBS-HYS-0017 | 0.001 | 0.999 | C2 |
| 31 | GBS-HYS-0018 | 0.068 | 0.932 | C2 |
| 33 | GBS-HYS-0019 | 0.138 | 0.862 | C2 |
| 34 | GBS-HYS-0020 | 0.083 | 0.917 | C2 |
| 35 | GBS-HYS-0021 | 0.998 | 0.002 | C1 |
| 36 | GBS-HYS-0022 | 0.054 | 0.946 | C2 |
| 38 | GBS-HYS-0023 | 0.092 | 0.908 | C2 |
| 40 | GBS-HYS-0024 | 0.098 | 0.902 | C2 |
| 41 | GBS-HYS-0025 | 0.011 | 0.989 | C2 |
| 43 | GBS-HYS-0026 | 0.127 | 0.873 | C2 |
| 44 | GBS-HYS-0027 | 0.133 | 0.867 | C2 |
| 45 | GBS-HYS-0028 | 0.001 | 0.999 | C2 |
| 47 | GBS-HYS-0029 | 0.112 | 0.888 | C2 |
| 52 | GBS-HYS-0030 | 0.105 | 0.895 | C2 |
| 53 | GBS-HYS-0031 | 0.142 | 0.858 | C2 |
| 55 | GBS-HYS-0032 | 0.268 | 0.732 | C2 |
| 57 | GBS-HYS-0033 | 0.057 | 0.943 | C2 |
| 61 | GBS-HYS-0034 | 0.629 | 0.371 | Admixt |
| 66 | GBS-HYS-0035 | 0.002 | 0.998 | C2 |
| 68 | GBS-HYS-0036 | 0.008 | 0.992 | C2 |
| 69 | GBS-HYS-0037 | 0.013 | 0.987 | C2 |
| 70 | GBS-HYS-0038 | 0.001 | 0.999 | C2 |
| 71 | GBS-HYS-0039 | 0.169 | 0.831 | C2 |
| 74 | GBS-HYS-0040 | 0.001 | 0.999 | C2 |
| 77 | GBS-HYS-0041 | 0.04 | 0.96 | C2 |
| 79 | GBS-HYS-0042 | 0.122 | 0.878 | C2 |
| 82 | GBS-HYS-0043 | 0.058 | 0.942 | C2 |
| 84 | GBS-HYS-0044 | 0.125 | 0.875 | C2 |
| 86 | GBS-HYS-0045 | 0.063 | 0.937 | C2 |
| 87 | GBS-HYS-0046 | 0.008 | 0.992 | C2 |
| 88 | GBS-HYS-0047 | 0.003 | 0.997 | C2 |
| 89 | GBS-HYS-0048 | 0.005 | 0.995 | C2 |
| 90 | GBS-HYS-0049 | 0.001 | 0.999 | C2 |
| 91 | GBS-HYS-0050 | 0.001 | 0.999 | C2 |
| 92 | GBS-HYS-0051 | 0.078 | 0.922 | C2 |
| 98 | GBS-HYS-0052 | 0.034 | 0.966 | C2 |
| 102 | GBS-HYS-0053 | 0.002 | 0.998 | C2 |
| 103 | GBS-HYS-0054 | 0.007 | 0.993 | C2 |
| 104 | GBS-HYS-0055 | 0.001 | 0.999 | C2 |
| 105 | GBS-HYS-0056 | 0.001 | 0.999 | C2 |
| 106 | GBS-HYS-0057 | 0.177 | 0.823 | C2 |
| 109 | GBS-HYS-0058 | 0.015 | 0.985 | C2 |
| 110 | GBS-HYS-0059 | 0.081 | 0.919 | C2 |
| 111 | GBS-HYS-0060 | 0.135 | 0.865 | C2 |
| 114 | GBS-HYS-0061 | 0.105 | 0.895 | C2 |
| 118 | GBS-HYS-0062 | 0.001 | 0.999 | C2 |
| 119 | GBS-HYS-0063 | 0.065 | 0.935 | C2 |
| 124 | GBS-HYS-0064 | 0.469 | 0.531 | Admixt |
| 126 | GBS-HYS-0065 | 0.175 | 0.825 | C2 |
| 127 | GBS-HYS-0066 | 0.999 | 0.001 | C1 |
| 129 | GBS-HYS-0067 | 0.033 | 0.967 | C2 |
| 130 | GBS-HYS-0068 | 0.174 | 0.826 | C2 |
| 132 | GBS-HYS-0069 | 0.243 | 0.757 | C2 |
| 134 | GBS-HYS-0070 | 0.28 | 0.72 | C2 |
| 135 | GBS-HYS-0071 | 0.069 | 0.931 | C2 |
| 137 | GBS-HYS-0072 | 0.609 | 0.391 | Admixt |
| 138 | GBS-HYS-0073 | 0.024 | 0.976 | C2 |
| 139 | GBS-HYS-0074 | 0.001 | 0.999 | C2 |
| 141 | GBS-HYS-0075 | 0.567 | 0.433 | Admixt |
| 143 | GBS-HYS-0076 | 0.161 | 0.839 | C2 |
| 144 | GBS-HYS-0077 | 0.057 | 0.943 | C2 |
| 147 | GBS-HYS-0078 | 0.011 | 0.989 | C2 |
| 151 | GBS-HYS-0079 | 0.929 | 0.071 | C2 |
| 152 | GBS-HYS-0080 | 0.247 | 0.753 | C2 |
| 153 | GBS-HYS-0081 | 0.097 | 0.903 | C2 |
| 155 | GBS-HYS-0082 | 0.001 | 0.999 | C2 |
| 157 | GBS-HYS-0083 | 0.179 | 0.821 | C2 |
| 158 | GBS-HYS-0084 | 0.817 | 0.183 | C1 |
| 159 | GBS-HYS-0085 | 0.003 | 0.997 | C2 |
| 164 | GBS-HYS-0086 | 0.225 | 0.775 | C2 |
| 165 | GBS-HYS-0087 | 0.147 | 0.853 | C2 |
| 166 | GBS-HYS-0088 | 0.008 | 0.992 | C2 |
| 167 | GBS-HYS-0089 | 0.001 | 0.999 | C2 |
| 168 | GBS-HYS-0090 | 0.163 | 0.837 | C2 |
| 170 | GBS-HYS-0091 | 0.122 | 0.878 | C2 |
| 171 | GBS-HYS-0092 | 0.093 | 0.907 | C2 |
| 172 | GBS-HYS-0093 | 0.058 | 0.942 | C2 |
| 173 | GBS-HYS-0094 | 0.015 | 0.985 | C2 |
| 177 | GBS-HYS-0095 | 0.076 | 0.924 | C2 |
| 178 | GBS-HYS-0096 | 0.444 | 0.556 | Admixt |
| 180 | GBS-HYS-0097 | 1 | 0 | C1 |
| 183 | GBS-HYS-0098 | 0.36 | 0.64 | Admixt |
| 184 | GBS-HYS-0099 | 0.566 | 0.434 | Admixt |
| 185 | GBS-HYS-0100 | 0.077 | 0.923 | C2 |
| 186 | GBS-HYS-0101 | 0.106 | 0.894 | C2 |
| 187 | GBS-HYS-0102 | 0.572 | 0.428 | Admixt |
| 189 | GBS-HYS-0103 | 0.002 | 0.998 | C2 |
| 191 | GBS-HYS-0104 | 0.023 | 0.977 | C2 |
| 194 | GBS-HYS-0105 | 0.086 | 0.914 | C2 |
| 195 | GBS-HYS-0106 | 0.133 | 0.867 | C2 |
| 198 | GBS-HYS-0107 | 0.75 | 0.25 | C1 |
| 199 | GBS-HYS-0108 | 0.329 | 0.671 | Admixt |
| 200 | GBS-HYS-0109 | 0.941 | 0.059 | C1 |
| 202 | GBS-HYS-0110 | 0.826 | 0.174 | C1 |
| 203 | GBS-HYS-0111 | 0.02 | 0.98 | C2 |
| 205 | GBS-HYS-0112 | 0.112 | 0.888 | C2 |
| 209 | GBS-HYS-0113 | 0.022 | 0.978 | C2 |
| 211 | GBS-HYS-0114 | 0.054 | 0.946 | C2 |
| 212 | GBS-HYS-0115 | 0.078 | 0.922 | C2 |
| 213 | GBS-HYS-0116 | 0.972 | 0.028 | C1 |
| 214 | GBS-HYS-0117 | 0.126 | 0.874 | C2 |
| 218 | GBS-HYS-0118 | 0.591 | 0.409 | Admixt |
| 219 | GBS-HYS-0119 | 0.024 | 0.976 | C2 |
| 220 | GBS-HYS-0120 | 0.001 | 0.999 | C2 |
| 222 | GBS-HYS-0121 | 0.086 | 0.914 | C2 |
| 224 | GBS-HYS-0122 | 0.036 | 0.964 | C2 |
| 225 | GBS-HYS-0123 | 0.072 | 0.928 | C2 |
| 226 | GBS-HYS-0124 | 0.129 | 0.871 | C2 |
| 227 | GBS-HYS-0125 | 0.995 | 0.005 | C1 |
| 228 | GBS-HYS-0126 | 0.38 | 0.62 | Admixt |
| 230 | GBS-HYS-0127 | 0.796 | 0.204 | C1 |
| 231 | GBS-HYS-0128 | 0.067 | 0.933 | C2 |
| 233 | GBS-HYS-0129 | 0.214 | 0.786 | C2 |
| 234 | GBS-HYS-0130 | 0.822 | 0.178 | C1 |
| 235 | GBS-HYS-0131 | 0.147 | 0.853 | C2 |
| 237 | GBS-HYS-0132 | 0.776 | 0.224 | C1 |
| 239 | GBS-HYS-0133 | 0.001 | 0.999 | C2 |
| 240 | GBS-HYS-0134 | 0.998 | 0.002 | C1 |
| 242 | GBS-HYS-0135 | 0.998 | 0.002 | C1 |
| 243 | GBS-HYS-0136 | 0.118 | 0.882 | C1 |
| 245 | GBS-HYS-0137 | 0.002 | 0.998 | C2 |
| 250 | GBS-HYS-0138 | 0.955 | 0.045 | C1 |
| 251 | GBS-HYS-0139 | 0.001 | 0.999 | C2 |
| 254 | GBS-HYS-0140 | 0.002 | 0.998 | C2 |
| 255 | GBS-HYS-0141 | 0.044 | 0.956 | C2 |
| 256 | GBS-HYS-0142 | 0.146 | 0.854 | C2 |
| 260 | GBS-HYS-0143 | 0.101 | 0.899 | C2 |
| 262 | GBS-HYS-0144 | 0.462 | 0.538 | Admixt |
| 264 | GBS-HYS-0145 | 0.167 | 0.833 | C2 |
| 265 | GBS-HYS-0146 | 0.02 | 0.98 | C2 |
| 266 | GBS-HYS-0147 | 0.043 | 0.957 | C2 |
| 268 | GBS-HYS-0148 | 0.8 | 0.2 | C1 |
| 269 | GBS-HYS-0149 | 1 | 0 | C1 |
| 270 | GBS-HYS-0150 | 0.171 | 0.829 | C2 |
| 271 | GBS-HYS-0151 | 0.021 | 0.979 | C2 |
| 275 | GBS-HYS-0152 | 0.003 | 0.997 | C2 |
| 276 | GBS-HYS-0153 | 0.314 | 0.686 | C2 |
| 277 | GBS-HYS-0154 | 0.148 | 0.852 | C2 |
| 279 | GBS-HYS-0155 | 0.069 | 0.931 | C2 |
| 282 | GBS-HYS-0156 | 0.003 | 0.997 | C2 |
| 283 | GBS-HYS-0157 | 0.165 | 0.835 | C2 |
| 285 | GBS-HYS-0158 | 0.074 | 0.926 | C2 |
| 286 | GBS-HYS-0159 | 0.044 | 0.956 | C2 |
| 289 | GBS-HYS-0160 | 0.127 | 0.873 | C2 |
| 290 | GBS-HYS-0161 | 0.13 | 0.87 | C2 |
| 291 | GBS-HYS-0162 | 0.078 | 0.922 | C2 |
| 292 | GBS-HYS-0163 | 0.109 | 0.891 | C2 |
| 293 | GBS-HYS-0164 | 0.006 | 0.994 | C2 |
| 295 | GBS-HYS-0165 | 0.074 | 0.926 | C2 |
| 296 | GBS-HYS-0166 | 0.08 | 0.92 | C2 |
| 297 | GBS-HYS-0167 | 0.102 | 0.898 | C2 |
| 298 | GBS-HYS-0168 | 0.003 | 0.997 | C2 |
| 299 | GBS-HYS-0169 | 0.087 | 0.913 | C2 |
| 300 | GBS-HYS-0170 | 0.038 | 0.962 | C2 |
| 301 | GBS-HYS-0171 | 0.131 | 0.869 | C2 |
| 303 | GBS-HYS-0172 | 0.095 | 0.905 | C2 |
| 304 | GBS-HYS-0173 | 0.003 | 0.997 | C2 |
| 307 | GBS-HYS-0174 | 0.304 | 0.696 | C2 |
| 309 | GBS-HYS-0175 | 0.058 | 0.942 | C2 |
