# Supplementary figures and images for: SNP markers revealed the genetic diversity and population structure of Mesosphaerum suaveolens (L.) Kuntze Syn. Hyptis suaveolens (L.) Poit accessions collected in Benin
Source: PLoS One. 2025 Sep 4;20(9):e0331702. doi: 10.1371/journal.pone.0331702 (PMC12410747; doi:10.1371/journal.pone.0331702)

# Delta K

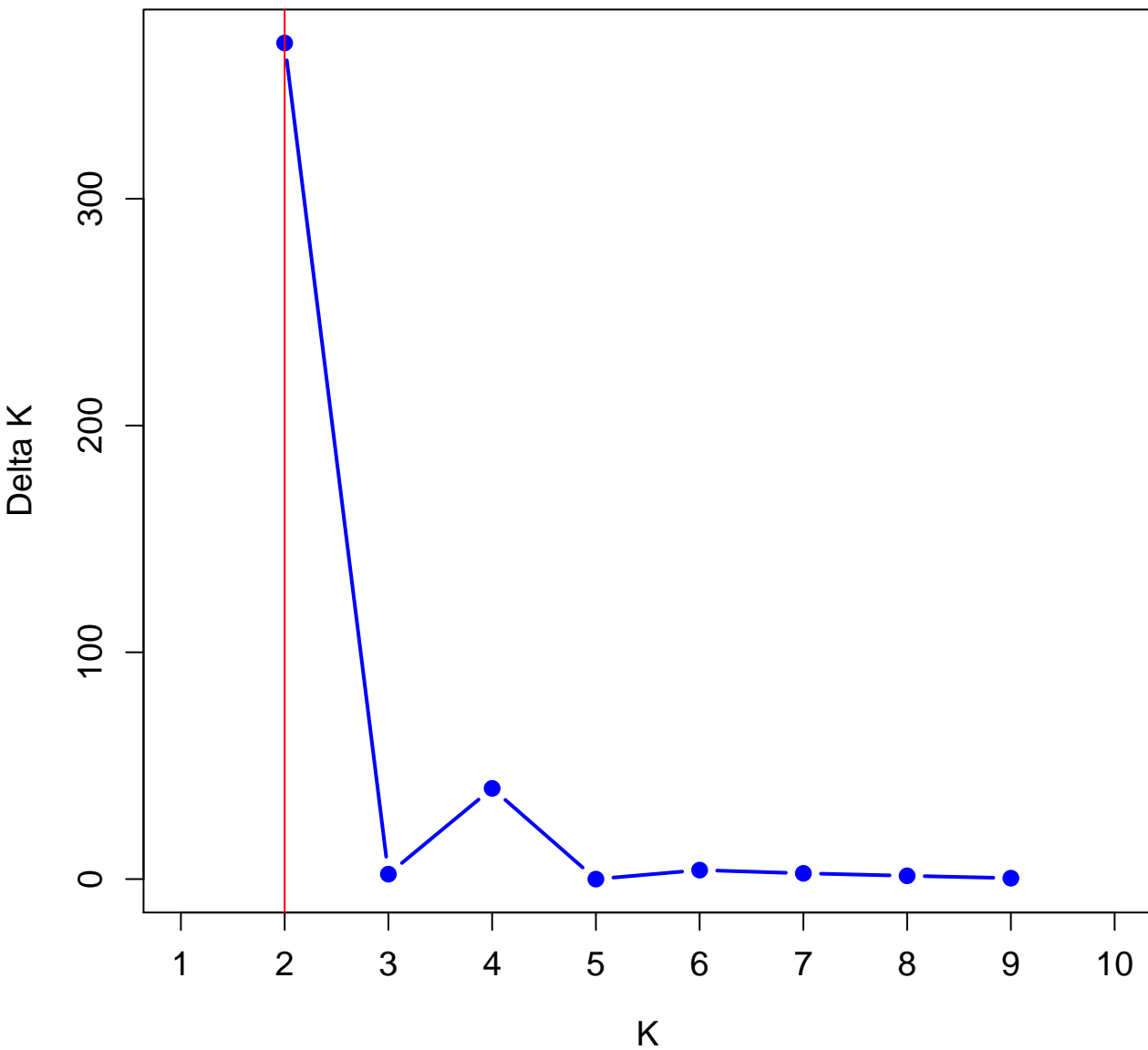

Supplement: S1 Fig — (PDF) [file pone.0331702.s003.pdf]
