# Supplementary material for: SNP markers revealed the genetic diversity and population structure of Mesosphaerum suaveolens (L.) Kuntze Syn. Hyptis suaveolens (L.) Poit accessions collected in Benin
Source: PLoS One. 2025 Sep 4;20(9):e0331702. doi: 10.1371/journal.pone.0331702 (PMC12410747; doi:10.1371/journal.pone.0331702)
Supplement: Attachment — Submitted filename: Response to reviewers.docx [file pone.0331702.s006.docx]

Manuscript ID: PONE-D-24-37428

Journal: PLOS ONE
Article type: Research Article

Manuscript title: SNP markers revealed the genetic diversity and population structure of *Mesosphaerum suaveolens* (L.) Kuntze Syn. *Hyptis suaveolens* (L.) Poit accessions collected in Benin

Authors: Armel Frida Dossa*, Dèdéou A. Tchokponhoué, Aristide C. Houdegbe, Enoch G. Achigan-Dako*

Responses to Editor and reviewers' comments

**Editor**

Journal requirement

Comment#1. Please ensure that your manuscript meets PLOS ONE's style requirements, including those for file naming.

Response #1*.*

We thank the reviewers for the suggestion. The manuscript meets PLOS ONE's style requirements, including file naming requirements.

Comment #2. We suggest you thoroughly copyedit your manuscript for language usage, spelling, and grammar. If you do not know anyone who can help you do this, you may wish to consider employing a professional scientific editing service.

Response #2.

We thank the reviewer for the suggestion. The Manuscript has been copyedited as requested.

Comment #3. Please note that PLOS ONE has specific guidelines on code sharing for submissions in which author-generated code underpins the findings in the manuscript. In these cases, we expect all author-generated code to be made available without restrictions upon publication of the work. Please review our guidelines at https://journals.plos.org/plosone/s/materials-and-software-sharing#loc-sharing-code and ensure that your code is shared in a way that follows best practices and facilitates reproducibility and reuse.

Response #3.

We thank the reviewer for the suggestion. No new codes were generated in this work.

Comment #4. In your Methods section, please provide additional information regarding the permits you obtained for the work. Please ensure you have included the full name of the authority that approved the field site access and, if no permits were required, a brief statement explaining why.

Response#4.

This work is part of the PhD research program of the first author. The authors defended her protocol in front of panel of the University lecturers and after approval systematically benefited from an approval from her fieldwork including samples collection. As such, no other permits were required to access the field.

Comment #5. Please include your full ethics statement in the ‘Methods’ section of your manuscript file. In your statement, please include the full name of the IRB or ethics committee who approved or waived your study, as well as whether or not you obtained informed written or verbal consent. If consent was waived for your study, please include this information in your statement as well.

Response #5.

We thank the reviewer for the suggestion. The ethics committee that waived the study is the first author’s thesis committee at the University of Abomey-Calavi. This committee is made up of Prof Enoch G. Achigan-Dako, Prof Eleonore Yayi-Ladekan, Prof Bokonon-Ganta Aimee, Prof Latifou Lagnikan, and Prof Victorien T. Dougnon. In addition, the informed verbal consent of all local authorities was obtained prior the collection of the samples. (Lines 198- 209)

Comment #6. we note that Figure 1 in your submission contain copyrighted images. All PLOS content is published under the Creative Commons Attribution License (CC BY 4.0), which means that the manuscript, images, and Supporting Information files will be freely available online, and any third party is permitted to access, download, copy, distribute, and use these materials in any way, even commercially, with proper attribution. For more information, see our copyright guidelines: http://journals.plos.org/plosone/s/licenses-and-copyright.
 
We require you to either (1) present written permission from the copyright holder to publish these figures specifically under the CC BY 4.0 license, or (2) remove the figures from your submission:

Response #6.

We thank the reviewer for the suggestion. The images in Figure 1 have been replaced by new images collected in the field.

Comment #7 We note that Figure 1 in your submission contains map/satellite image which may be copyrighted. All PLOS content is published under the Creative Commons Attribution License (CC BY 4.0), which means that the manuscript, images, and Supporting Information files will be freely available online, and any third party is permitted to access, download, copy, distribute, and use these materials in any way, even commercially, with proper attribution. For these reasons, we cannot publish previously copyrighted maps or satellite images created using proprietary data, such as Google software (Google Maps, Street View, and Earth). For more information, see our copyright guidelines: http://journals.plos.org/plosone/s/licenses-and-copyright.

Response #7.

Our image 1 does not present any map/satellite.

Comment #8 We note that Figure 2 in your submission contain map/satellite image which may be copyrighted. All PLOS content is published under the Creative Commons Attribution License (CC BY 4.0), which means that the manuscript, images, and Supporting Information files will be freely available online, and any third party is permitted to access, download, copy, distribute, and use these materials in any way, even commercially, with proper attribution. For these reasons, we cannot publish previously copyrighted maps or satellite images created using proprietary data, such as Google software (Google Maps, Street View, and Earth). For more information, see our copyright guidelines: <http://journals.plos.org/plosone/s/licenses-and-copyright>.

Response #8.

The figure 2 has been deleted.

**Reviewer #1**

Comment #1.

This work would be more complete if you have incorporated data on those important traits you mentioned in the manuscript. After all, genetic diversity is relevant to breeders and conservationists. For breeder the molecular diversity has to be supported by morphological data where he can see if the diversity is functional and because of certain traits (past artificial or natural selection). Otherwise if the diversity is mainly because of SNPs that have no agronomic importance, it will have no use for the breeder. Hence I strongly recommend to incorporate data on a few traits and see after doing PCA or NJ-tree to see if those traits are determinants of population structure and differentiation.

Response #1.

We thank the reviewer for his positive suggestions. Although his comment is relevant, we would like to indicate that the focus of this current paper is to gauge the extent of diversity in the species controlling for confounding effects morphological traits may bring to environmental noises. In addition, we also had another full paper on the agromorphological characterization of the species that is currently under review in another journal. Given these considerations, we think that we are happy with only presenting SNPs in this current manuscript.

**Reviewer #2**

Comment #1. It is better to use the recent references

Response #1.

We thank the reviewer for the suggestion. Some recent references have been added to the Manuscript. This includes the citation numbers: 12,13,14,15,16,17,18,19,20,21,22,23,26,27,30,33, 52,53,54,55, 57,58,59,60

(Line 56-86).

Comment #2. Detailed information regarding the genetic diversity and its varieties should be included.

Response #2.

We thank the reviewer for the suggestion. Information regarding genetic diversity and its varieties was clearly emphasized in the introduction (Lines 80-86; 99- 106). Polymorphic alleles information has been now added (Line 112-127

Comment #3. Detailed information regarding the genetic diversity methods should be included

Response #3.

We thank the reviewer for the suggestion. Detailed information regarding the genetic diversity methods have now been included throughout the manuscript (Lines 127-146).

Comment #4. It would be beneficial to include a few lines regarding the plant species' beneficial applications.

Response #4.

We thank the reviewer for that suggestion. The beneficial applications have been included and some references and examples have been provided to support *M. suaveolens* essential oils potential for agriculture and medicinal applications (Line 60-76).

“ Oils were effective in controlling *Sitophilus zeamais* (Motschulsky) (Coleoptera: Curculionidae), *Maruca vitrata* (Fabricius) (Lepidoptera: Crambidae), *Megalurothrips sjostedti* (Trybom) (Thysanoptera: Thripidae); and *Aphis craccivora* Koch (Hemiptera: Aphididae)”;

“It is also considered as eco-friendly herbicide due to its germination, and seedling growth inhibition effect on weeds including *Cereus jamacaru*. DC. Subsp. Jamacaru (Cactaceae), and *Echinochloa. crus-galli* (L.) P.Beauv. (Poaceae) associated with rice”;

“Its roots, leaves, and twigs are used as decoction to heal several ailments, including digestive infections, malaria, skin infections, constipation, renal inflammation, headaches, and respiratory issues injuries (6,15,20,24,25). The oils exhibited anti-cancer properties against human breast cancer, and possess anti-inflammatory properties”

Comment #5. Why did the researchers select this plant species?

Response #5.

We are grateful to the reviewer for posing the question. This species was selected for study due to its status as a wild aromatic plant with many beneficial functions for both humans and the surrounding environment. *M. suaveolens* demonstrates a high reproductive capacity. The biological effect of its highly variable essential oil has been demonstrated on fungi, viruses, bacteria, and various insect pests of crops of economic interest. It is recognized as a biopesticide, which presents a solution for preserving humanity and the natural world in the context of climate change. Despite the benefits it offers in medicine and agriculture, it is not yet employed on a large scale in real-world environments, one of the reasons being its low oil yield. Furthermore, despite its significance, the genetic diversity of this species has been insufficiently investigated, even though the variability of the chemical composition of its essential oil and its oil yield depend on the genotypes, and therefore on the existing diversity. In general, this research seeks to generate molecular information about *M. suaveolens* to facilitate breeding research efforts for improved yield and chemical content. (Line 60-76; 87-151)

Comment #6. More information about the used markers should be stated

Response #6.

We thank the reviewer for that suggestion. Some informations and references have been added to the manuscript about DArTseq technology. (Line 136-146)

‘’DArTseq technology generates a large pool of high-quality SNPs and SilicoDArT marker, even in species without a reference genome. Furthermore ; DArTseq is an excellent approach for genotyping a wide number of individuals (48,54,55).It has been used to reveal the genetic diversity and population structure of 62 aromatic basils (*Ocimum basilicum*) from Lamiaceae family in Ethiopia (55). Numerous molecular studies have used DArTseq to explore the genetic diversity, to identify marker of interest, or to assess the relationship in species lines for several crops such as *Vigna inguiculata* (54), *Colocasia esculenta* (56), *Synsepalum dulcificum* (57), Zea mays (58), *Phaseolus vulgaris* (59), *Musa acuminata* (60) and *Macrotyloma geocarpum*(60) ‘’

Comment #7. The hypothesis is inaccurately articulated. The authors ought to incorporate a brief section delineating the knowledge gaps they sought to address in their research.

Response #7.

We thank the reviewer for that suggestion. We have now added this additional paragraph delineating knowledge gaps (Line 80-86).

“The results of *M. suaveolens* essential oil application as a biopesticide are still at the laboratory stage. Its large-scale use in a real environment is hampered by its low essential oil yield. Since variability in essential oil chemical composition and yield is linked to genotype and environment, studies of agromorphological variability and genetic diversity within the species could help to overcome this challenge by identifying genotypes with high oil yield and quality (35).”

The hypothetis has been improved by adding a research hypothesis related to population structure. (Line 164-166)

Comment #8. Furthermore, authors must provide a statement emphasizing the uniqueness of their findings in the conclusion.

Response #8.

We thank the reviewer for that suggestion. The uniqueness of the study was stressed on in the the introduction. (See Lines 148 – 151, revised version)

“Studies of genetic diversity within the species based on single-nucleotide polymorphisms have not been reported in this species worldwide, and this is the gap that our study integrating samples from various phytogeographical regions of Benin is filling”.

The uniqueness of the study was stressed on in the first sentence of the conclusion. (See Lines506– 508, revised version)

‘‘ Worldwide, this study is the first to describe for the first time the population structure and genetic diversity in *M. suaveolens* using SNP, thus setting the scene for a proper marker-assisted improvement of useful traits in the species.’’

Comment #9.. What novel research-related connections or activities have the authors identified in this study that were absent in prior studies?

Response #9.

We thank the reviewer for that suggestion. As mentioned in the introduction, very few genetic diversity studies have been conducted on *M suaveolens*. The only known diversity study to date is that carried out by Gadidasu et al in 2011 using ISSR molecular markers. Apart from this study, none of the studies in the literature have assessed genetic diversity within the species. The novelty of our study is the assessment of diversity using new techniques that integrate a large number of individuals. This enables the generation of the first SNP markers from *M. suaveolens.*

Comment #10. It is imperative to address both the general and specific objectives.

Response #10.

We thank the reviewer for that suggestion. The objective of this study was to assess the extent of genetic diversity and population structure in *M. suaveolens* germplasm from Benin using high-density SNP markers, and we did not break down this. We hear the reviewer misunderstood us on this comment.

Comment #11. map showing the samples collection should be included

Response #11.

We thank the reviewer for that suggestion. The map of collection sites was included in the submitted manuscript. See the Fig 2. Map of *Mesosphaerum suaveolens* accessions collection sites in the three phytogeographical regions of Benin. (Lines 170;175)

Comment #12. The voucher number of this plant species should be provided

Response #12.

We thank the reviewer for that suggestion. A total of 175 accessions of the species was collected, and each of them has its own voucher number now added to the Table S1.

Comment #13. It is necessary to provide the entire name of each acronym.

Response #13.

We thank the reviewer for that suggestion. The entire name of each acronym has been integrated in the manuscript.

“International Livestock Research Institute (ILRI)” (Line 197)

“Diversity Arrays Technology and Sequencing (DarTseq)” (Line 215),

“Markov Chain Monte Carlo (MCMC)” (Line 245),

“The overall genetic fixation index (Fstg)”(Line 295)

Comment # 14. References must be provided to substantiate each technique.

Response #14.

We thank the reviewer for that suggestion. References have been added to the cited technique in the manuscript. (Lines 210-256)

Comment #15. The researchers should be performed the unique bands, genetic and diversity index for each marker and population

Response #15.

We thank the reviewer for that suggestion. We do not see it as relevant. The markers used here is an advanced type marker and no band scoring is necessary. This is a SNP-based study not a SSR marker-based one.

Comment #16. The manufacture name of all materials should be added

Response #16.

We thank the reviewer for that suggestion. Added where relevant. (Line 211)

Comment #17. The data analysis section should be located at the end of MM

Response #17.

This suggestion does not align with the PLoS One instructions. So, we stick to the current structure.

Comments #18. Captions should be improved. It is necessary to include definitions for all abbreviations in the captions of tables and figures.

Response #18.

We thank the reviewer for that suggestion. Definitions for all abbreviations have been added in the captions of tables and figures (Lines 279; 289; 318)

Comment # 19.. Creating a PCA plot should be provided

Response# 19.

We thank the reviewer for that suggestion. A PCA will not bring any added value to the information already presented since we have already presented a phylogenetic tree as well as structure analysis that are better suited to answer our research questions.

Comment #20.. The dendrogram should be annotated with the number of clusters.

Response #20.

We thank the reviewer for that suggestion. The number of clusters has been added.

Comment #21. What are the criteria used to determine the number of clusters?

Response #21.

We thank the reviewer for that question. The average silhouette method was used. At the appropriate K-value, the distribution of likelihood values of K exhibits a notable inflection point, which is discernible in the Delta K-values. The illustrative graphic is presented as a supplementary file (S1 Fig) (lines 247-249)

Comment #22. The discourse lacked conviction. The commentary is insufficient and necessitates updating, as most phrases reiterate the findings instead of offering a critical examination of the data. The authors must elucidate the importance of the findings from each study in connection to their own research. The authors are accountable for elucidating the findings about the diversity index, clustering, PIC, and He. The authors ought to elucidate the disparity in the accumulation of bioactive across various areas.

Response#22.

We thank the reviewer for that suggestion. The Discussion has been carefully improved in the light of the reviewer’s relevant comments (Lines 346 - 484).

- PIC

“This informativeness is similar to that reported in the Ethiopian *Ocimum basilicum* study which varied from 0.23 to 0.37 using DArTseq (54). Overall, the markers used were informative enough to help appreciate the extent of diversity in the species. The moderate PIC value of the SNP markers in this study may be compensated by the large number of markers and the wide genome coverage (58).”

- Expected heterozygosity (*He*) (Lines 361-384)

“The expected heterozygosity (*He*) presented an average value of 0.287, indicating low genetic diversity in the studied *M. suaveolens* germplasm. This result corroborates the results of Gossa et al. (54) for a population of *O*. *basilicum*, which presented low diversity (*He* varied from 0.13 to 0.33) among the accessions evaluated using SNP markers. Kyrkjeeide et al.(78) also reported a total expected heterozygosity (*He* = 0.27) relatively similar to our result using SNP markers within populations of *Dracocephalum ruyschiana*, an aromatic plant from the Lamiaceae family (78). Wild species such as *M. suaveolens*, which are widely distributed, are expected to exhibit a relatively high level of diversity. Nevertheless, the low genetic diversity recorded in this study may be attributed to various factors. First, the geographical coverage of the study, although encompassing three clearly demarcated ecological regions of Benin, is still limited to the Dahomey Gap, which is known as a single ecology in the African rainforest space. We then speculated that expanding the collection areas could increase the diversity level (79). It is therefore imperative to undertake efforts to collect and preserve the genetic diversity of *M. suaveolens* present in Benin and worldwide. Second, the species was previously reported to exhibit both autogamy and allogamy, and the low diversity observed might represent a signal of the preponderance of autogamy in the species. This was experimentally confirmed by Aluri and Reddi (80). In addition, in Benin, *M. suaveolens* species are facing landscape fragmentation, a phenomenon known to reduce the effective size of wild populations and the variation of diversity (76). Faced with this phenomenon, the assessment of genetic diversity are fundamental studies that help to select worthwhile genotypes to be used for future biological research studies. Genetic variability of aromatic genotypes within the same species can instigate the production of numerous active chemical constituents (33,77).

The low diversity in the present study coupled with a moderate inbreeding coefficient (*F*_IS_ = 0.59), indicating a moderate level of fixation. This value of inbreeding could also justify both autogamy and allogamy in the species (10). However, the Sudanian population appeared to be the most diverse of the three studied populations, suggesting that it may have more potential to adapt to the changing climate in a region already known to experience severe climatic conditions. Therefore, this population is a potential source of genes of interest that could be deployed in improvement programs for the development of specific product profile genotypes. Hence, there is urgent need for more concentrated actions towards collecting genetic resources throughout the region for ex-situ conservation strategy profiling. Using SNP markers allowed us to evaluate the relationships for new future breeding or conservation efforts. Fst measures a population’s differentiation linked to genetic structure (81). An Fst value > 0.25 indicates very high differentiation, an Fst < 0.05 implies low differentiation (82). The values ranging from 0.05 to 0.15 and 0.15 to 0.25 reflected moderate and high differentiation, respectively. However, in this study’s Fst value (Fstg = 0.007) observed suggests very low genetic differentiation between these three populations in contrast to the findings of Salifou et al (17) who reported two chemotypes of *M. suaveolens* according to the three phytogeographical regions of Benin, β-caryophyllene (Sudanian and Sudano-guinean), and 1,8-cineole (Guineo-congolian) which is somewhat predictive of genotype grouping based on accession’s location. However, the present low degree of differentiation between populations of *M. suaveolens* aligned well with the results of AMOVA (Table 4), where the greatest proportion of genetic variation was explained by differences among samples within populations. The results indicate a high degree of genetic variation among accessions within each geographic origin, with those from different regions exhibiting minimal divergence. This finding corroborates the cluster analysis, which demonstrated that the accessions did not form distinct clusters based on their areas of collection/phytogeographical regions. Similar result was reported in the genetic diversity study of *Ocimum basilicum* which revealed that only 1% of the molecular variation was due to variations among populations (54). A low genetic differentiation among populations was reported for many other species from the Lamiaceae family including *Dracocephalum ruyschiana* and *Dracocephalum austriacum* (71,77). The differentiation observed in this study may also be associated with geographic origins or distance among the studied populations. As reported in Table 3, the closer the populations are, the smaller their genetic distances are, and the further apart they are, the greater their genetic distances are. Higher genetic differentiation observed between Sudanian and Guineo-congolian zones could be due to the great distance between the two regions. This result is comparable to those found by Akohoué et al (60) while evaluating genetic diversity in *Macrotyloma geocarpum* in Benin. Our study’s Fst value also indicates probable high gene flow among populations. Gene flow, which enhances genetic diversity within populations and reduces diversity among populations, is a factor affecting the genetic differentiation of populations, which is contrary to the effect of genetic drift (84). According to Yao et al (85), the low differentiation noted within cowpea populations is the consequence of long-distance gene dispersal, whether by pollen or by seed. This gene dispersal could also explain the low differentiation noted in this study as the sexual reproduction in *M. suaveolens* is possible through wind and insects contribution (80)

Differences in population structure display genetic variation and demonstrate a species’ capability to adjust to its changing habitat (86). The neighbor -joining analysis detected 3 clusters, whereas the STRUCTURE analysis revealed 2 genetic groups. This difference can be partly explained by the restriction of the software STRUCTURE to properly handle population structuring (60). In addition to this limitation, the results of STRUCTURE are sensitive to the marker type, population number, sample size, and number of loci scored (70). Similar mismatching results between neighbor-joining and structure analysis were reported by Akohoué et al (60) and Tchokponhoué et al (56) while structuring Kersting’s groundnut and the Sisrè berry populations respectively. Moreover, the Bayesian STRUCTURE analysis produced two groups, and 09.71% of the 175 *M. suaveolens* accessions were admixed. Pyne et al. (87) reported a 15.6% admixture between the 180 *Ocimum* spp. accessions studied. This level of admixture could further support the assumption of genomic content exchange, possibly during cross-pollination (87).This phenomenon was also reported in *Elsholtzia stauntonii* Benth. (Lamiaceae) (79). The results of the present study indicate that natural outcrossing has preserved some level of genetic diversity within *M. suaveolens* accessions. Furthermore, some accessions collected in the same districts were structured into different clusters. Natural selection, genetic mutation, and migration could be the main mechanism of evolution that affect these genetic differences (88). Based on the neighbor-joining tree, 77.8% of the sampled accessions were grouped into a single cluster (comprising both blue flowering and light purple flowering *M. suaveolens*). The accessions in this group were collected from heterogeneous fields and diverse habitats, including mountains, easy-to-access areas, and abandoned fields (89), where anthropic activities and animals are permanent. The species' habitat was mostly found along the road. *Mesosphaerum suaveolens* produces abundant and light, small seeds that can be easily transported without difficulty by water and wind over long distances. This could explain the high gene flow observed among populations and accessions with the evidence of the similitude found among accessions.

Intensive germplasm collection across different regions of the world is needed to determine the population divergence of this species. Considering the low genetic diversity within *M. suaveolens*, broadening the genetic base within the species could be helpful for future improvement programs. This will require cross-pollination among samples since the highest proportion of observed genomic variation resulted from variations among samples within populations. Furthermore, new germplasms need to be introduced, and genotypes from different continents and countries need to be crossed to increase the genetic diversity of *M. suaveolens*.”

Comment#23. The researchers ought to provide a concise summary of the most significant findings, as this section is presented in an easily understandable format. Future research should incorporate additional investigation on this subject.

Response#23.

We thank the reviewer for that suggestion. The conclusion has been improved by highlighting the more significant results and future ways of investigation. (Line 487-508)

“This SNP-based characterization study generated the first wide genetic polymorphism information in *M. suaveolens* using a high-throughput platform. This study provides insight into the population structure and genetic diversity of *M. suaveolens* in Benin. In total, 3,613 SNP markers showing minor allele frequency ≥ 0.5 were discovered. The analysis using the SNP markers revealed low genetic diversity within *M. suaveolens* accessions collected in Benin and lowly differentiated populations. The genetic diversity analysis showed two clusters among the studied accessions, with a high percentage (09.7%) of admixture. The genetic diversity was not structured according to geographic location/phytogeographical regions. The Sudanian population was more diverse than the Guineo-congolian and Guineo-sudanian populations. The discovered DArTseq SNP markers represent a novel genomic resource to improve *M. suaveolens* population management. These findings provide insights into understanding of genetic diversity and population structure of *M. suaveolens* accessions from three centres of diversity regions in Benin which could be exploited in the further genomics based studies, efficient genetic resource management, and in breeding program for genetic improvement for desirable product profile traits such as; essential oil yield and quality, and biosynthesis of some important pesticidal bioactive compounds. However, further investigations are needed regarding the combination of morphological, chemical constituents, and molecular analysis on one hand, and the genome assembly of *M. suaveolens* to allow genome-wide association studies (GWAS) on another hand. Worldwide, this study is the first to describe the population structure and genetic diversity in *M. suaveolens* using SNP, thus setting the scene for a proper marker-assisted improvement of useful traits in the species.

”
