# Supplementary material for: SNP markers revealed the genetic diversity and population structure of Mesosphaerum suaveolens (L.) Kuntze Syn. Hyptis suaveolens (L.) Poit accessions collected in Benin
Source: PLoS One. 2025 Sep 4;20(9):e0331702. doi: 10.1371/journal.pone.0331702 (PMC12410747; doi:10.1371/journal.pone.0331702)
Supplement: Attachment — Submitted filename: PLOS review_ Mesosphaerum.docx [file pone.0331702.s005.docx]

SNP markers revealed the genetic diversity and population structure of Mesosphaerum suaveolens (L.) Kuntze Syn. Hyptis suaveolens (L.) Poit accessions collected in Benin

I congratulate the authors upon this achievement. The results will inform the breeding and conservation of the species, so it has scientific merit. The data analysis is also okay. However, the introduction needs to be re-written for clarity and flow. The entire manuscript needs to be re-read and edited to minimize grammatical errors. Some specific comments are also indicated below.

L17-replace ‘genomic’ with ‘genetic’

L18-20 – For clarity, this sentence could be edited to ‘This research used single nucleotide polymorphism genotyping to study the diversity and population structure of ‘

L22-23-For clarity you could rewrite to ‘Based on the markers, a mean polymorphism information content of 0.28, a low….’

L29-30 -add the genetic diversity values

L33-35 needs clarity..Possibly you mean ‘However, genome-wide association studies (GWAS) to associate genomic and morphological or chemical constituents are needed’

L49-50-The flow is not smooth. I suggest that the information on chemical diversity is moved to after paragraph 73-81

L103-104 provide a citation

L118-add ‘s’ to marker

L120-add ‘s’ to marker. They are SNP markers

L121-change essays to assays

L128-add ‘s’ to marker

L129-instead put a comma after furthermore

L133-add ‘s’ to marker and check throughout the document

L138-139- edit the statement for clarity

L159- please explain why you used 10 m. For most out-crossing species, 100 m is normally used to minimize sampling of similar individuals

L160-delete the second ‘the’

L194-delete extra brackets after agarose

L261-265-I suggest that you use a Venn diagram for easy comprehension

L283-285. Overall, there is virtually no genetic differentiation. I hope this is well reflected in the discussion.

L332-342 has already been presented in the background. I suggest that you delete

L412-413 is misleading since there is virtually no genetic differentiation among all the 3 populations

L453-454..the low genetic differentiation could indicate high gene flow. Please explain how cross pollination can be helpful in enhancing diversity in this case.

The conclusion should be re-written to highlight the major take-home messages, not just repeating the results.
