# Supplementary material for: SNP markers revealed the genetic diversity and population structure of Mesosphaerum suaveolens (L.) Kuntze Syn. Hyptis suaveolens (L.) Poit accessions collected in Benin
Source: PLoS One. 2025 Sep 4;20(9):e0331702. doi: 10.1371/journal.pone.0331702 (PMC12410747; doi:10.1371/journal.pone.0331702)
Supplement: Attachment — Submitted filename: Response_to_reviewers_auresp_2.docx [file pone.0331702.s007.docx]

**Manuscript ID: PONE-D-24-37428**

**Journal: PLOS ONE**

**Article type: Research Article**

**SNP markers revealed the genetic diversity and population structure of *Mesosphaerum suaveolens* (L.) Kuntze Syn. *Hyptis suaveolens* (L.) Poit accessions collected in Benin**

Armel Frida Dossa*, Dèdéou A. Tchokponhoué, Aristide Carlos Houdegbe, Enoch G. Achigan-Dako*

**Responses to reviewers' comments**

**Reviewer #2**

**Comment #2.1.** Too many references under 2020 are used in this manuscript. Please minimize the number of references and the authors should use the recent references

**Response #2.1.** We thank the reviewer for this recommendation. In fact, the species is really under-studied, explaining why many references under 2020 were used. However, references under 2020 were minimized by removing some references (a total of 21) and more recent references were added. Please see the **References section**.

- ***Deleted references***

2.	Shenoy C, Patil MB, Kumar R. Wound healing activity of Hyptis suaveolens (L.) Poit (Lamiaceae). Int J Pharm Tech Res. 2009;1(3):737‑44.

3.	Bir SS. In IOPB chromosome number reports LXV. Taxon. 1979;28:630‑1.

5.	Krishnappa DG. IOPB chromosome number reports. LXXV. Taxon. 1982;31:342‑68.

6.	Jesus NZT, Falcão HS, Lima GRM, Caldas Filho MRD, Sales IRP, Gomes IF, et al. Hyptis suaveolens (L.) Poit (Lamiaceae), a medicinal plant protects the stomach against several gastric ulcer models. Journal of ethnopharmacology. 2013;150(3):982‑8.

9.	Thomford E Nicholas, Dzobo K, Adu F, Chirikure S, Wonkam A, Dandara C. Bush mint (Hyptis suaveolens) and spreading hogweed (Boerhavia diffusa) medicinal plant extracts differentially affect activities of CYP1A2, CYP2D6 and CYP3A4 enzymes. Journal of Ethnopharmacology. 30 janv 2018;211:58‑69.

12.	Azevedo NR, Campos IFP, Ferreira HD, Portes TA, Santos SC, Seraphin JC, et al. Chemical variability in the essential oil of Hyptis suaveolens. Phytochemistry. 1 juill 2001;57(5):733‑6.

16.	Noudegbessi AM, Alabi OY, Sikirou R. Olfactory responses of Sitophilus zeamais L. to bushmint leaf powder and methanol extract on stored maize. African Crop Science Journal. 2021;29(1):1‑12.

19.	Adamou M, Kosini D, Tchoubou-Salé A, Massah OD, Tchocgnia TFC, Mohammadou M, et al. Impact of aqueous extracts of Cassia occidentalis, Eucalyptus camaldulensis and Hyptis suaveolens on the entomofauna and the seed yield of Gossypium hirsutum at Boklé (Garoua, Cameroon). Heliyon. 1 oct 2022;8(10):e10937.

20.	dos Santos KP, Sedano-Partida MD, Sala-Carvalho WR, Loureiro BOSJ, da Silva-Luz CL, Furlan CM. Biological activity of Hyptis Jacq. (Lamiaceae) is determined by the environment. Industrial Crops and Products. 1 févr 2018;112:705‑15.

21.	Johnson F, Oussou KR, Kanko C, Tonzibo ZF, Foua-Bi K, Tano Y. Bioefficacite des Huiles Essentielles de Trois Especes Vegetales (Ocimum Gratissimum, Ocimum Canum et Hyptis Suaveolens), de la Famille des Labiees dans la Lutte Contre. European Journal of Scientific Research. oct 2018;150(3):12.

23.	Shubhashree KR, Reddy R, Gangula AK, Nagananda GS, Badiya PK, Ramamurthy SS, et al. Green synthesis of copper nanoparticles using aqueous extracts from Hyptis suaveolens (L.). Materials Chemistry and Physics. 2022;280:125795.

32.	Limachi I, Condo C, Palma C. Antiparasitic metabolites from plants used in the Tacana Bolivian native tribe: Traditional medicine, an overview of natural products with medicinal interest. Nat Prod Commun. 2019;14:55‑8.

34.	Tulugu M, Sujatha B, Sitalaxmi B. Anatomical, phytochemical and in vitro antimicrobial studies of Hyptis suaveolens L. Poit. of family Lamiaceae. International Journal of Bio-Pharma Research. 2019;8(6):2617‑22.

38.	Vieira R, Grayer R, Paton A, Simon J. Genetic diversity of Ocimum gratissimum L. Based on volatile oil constituents, flavonoids and RAPD markers. Biochemical Systematics and Ecology. 1 avr 2001;29:287‑304.

41.	Lal RK. Breeding for new chemotypes with stable high essential oil yield in Ocimum. Industrial Crops and Products. 2014;59:41‑9.

42.	Rastogi S, Kalra A, Gupta V, Khan F, Lal RK, Tripathi AK, et al. Unravelling the genome of Holy basil: an “incomparable”“elixir of life” of traditional Indian medicine. BMC genomics. 2015;16(1):1‑15.

43.	Ibrahim H, Salama AM, Abou El-Leel OF. Analysis of genetic diversity of Lavandula species using taxonomic, essential oil and molecular genetic markers. Sciences. 2017;7(01):141‑54.

45.	Wulff R. Intrapopulational variation in the germination of seeds in Hyptis suaveolens. Ecology. 1973;54(3):646‑9.

47.	Reddy ChintallaSR, Bagyanarayana G, Reddy KN. Invasive alien flora of India. 1 mars 2008;

51.	Zhang J, Singh A, Mueller DS, Singh AK. Genome‐wide association and epistasis studies unravel the genetic architecture of sudden death syndrome resistance in soybean. The Plant Journal. 2015;84(6):1124‑36.

53.	Elshire RJ, Glaubitz JC, Sun Q, Poland JA, Kawamoto K, Buckler ES, et al. A robust, simple genotyping-by-sequencing (GBS) approach for high diversity species. PloS one. 2011;6(5):e19379.

54.	Kethom W, Tongyoo P, Mongkolporn O. Genetic diversity and capsaicinoids content association of Thai chili landraces analyzed by whole genome sequencing-based SNPs. Scientia Horticulturae. 30 avr 2019;249:401‑6.

55.	van Deventer R, Rhode C, Marx M, Roodt-Wilding R. The development of genome-wide single nucleotide polymorphisms in blue wildebeest using the DArTseq platform. Genomics. 1 sept 2020;112(5):3455‑64.

- ***Added recent references***

21.	da Silva ST, Coelho AD, de Assis RMA, de Carvalho AA, Bertolucci SKV, Pinto JEBP. Influence of light quality and some growth regulators in inducing the production of Podophyllotoxin, a bioactive compound against cancer, in adventitious roots formed in the leaves of *Hyptis suaveolens*. Industrial Crops and Products. 15 nov 2022;188:115710.

69.	Francini A, Pintado M, Manganaris GA, Ferrante A. Editorial: Bioactive Compounds Biosynthesis and Metabolism in Fruit and Vegetables. Front Plant Sci [Internet]. 19 févr 2020 [cité 4 mai 2025];11. Disponible sur: https://www.frontiersin.orghttps://www.frontiersin.org/journals/plant-science/articles/10.3389/fpls.2020.00129/full

67.	Coelho AD, de Assis RMA, Leite JJF, Rocha JPM, Bertolucci SKV, Pinto JEBP. Podophyllotoxin bioaccumulation with biotic and abiotic elicitors on root culture of Hyptis suaveolens (L.) POIT. In Vitro CellDevBiol-Plant. 1 févr 2025;61(1):51‑66.

68.	Coelho AD, Mendonça SC, Assis RMA de, Rocha JPM, Shukla MR, Saxena PK, et al. Impact of phloroglucinol and indoleamines on growth and accumulation of podophyllotoxin in the roots of Hyptis suaveolens (L.) Poit. Plant Cell Tiss Organ Cult. 27 août 2024;158(3):50.

70.	Shahhat IMAM, Elsheikh SYS. Accumulation of secondary metabolites in the family Lamiaceae as influenced by foliar micronutrients. Boletín Latinoamericano y del Caribe de Plantas Medicinales y Aromáticas. 7 juill 2024;23(6):823‑38.

**Comment #2.2**. The conclusion is too long and should be summarized.

**Response #2.2.** The conclusion was revised (**see Line 536-561 of the revised manuscript with track changes**).

**Comment #2.3.** The discussion needs an improvement.

**Response #2.3.** The discussion section was revised for improvement (**see Lines 363-534 of the revised manuscript with track changes**).

**Reviewer #3**

**Comment #3.1.** The results will inform the breeding and conservation of the species, so it has scientific merit. The data analysis is also okay. However, the introduction needs to be re-written for clarity and flow. The entire manuscript needs to be re-read and edited to minimize grammatical errors.

**Response #3.1.** We thank the reviewer for this recommendation. The Introduction was re-written for clarity and flow (**See lines 42-183 of the revised manuscript with track changes**). Furthermore, the entire manuscript was re-read and edited to correct grammatical errors (See throughout **the revised manuscript with track changes)**.

**Comment #3.2**. L17-replace ‘genomic’ with ‘genetic’

**Response #3.2.** ‘genomic’ was replaced by ‘genetic’ (**Line 17** **of the revised manuscript with track changes**).

**Comment #3.3.** L18-20 – For clarity, this sentence could be edited to ‘This research used single nucleotide polymorphism genotyping to study the diversity and population structure of’.

**Response #3.3.** This correction was made. **(Lines 19-21 of the revised manuscript with track changes)**

**Comment #3.4.** L22-23-For clarity you could rewrite to ‘Based on the markers, a mean polymorphism information content of 0.28, a low….’

**Response #3.4.** The previous phrase were replaced by ‘Based on the markers, a mean polymorphism information content of 0.28, a low….’ (**Lines 23-24 of the revised manuscript with track changes**).

**Comment #3.5.** L29-30 -add the genetic diversity values

**Response #3.5.** The genetic diversity values were added **(Lines 30-32 of the revised manuscript with track changes)**.

**Comment #3.6.** L33-35 needs clarity. Possibly you mean ‘However, genome-wide association

studies (GWAS) to associate genomic and morphological or chemical constituents are needed’

**Response #3.6.** The previous sentence was replaced by ‘However, genome reference and genome-wide association studies (GWAS) to associate genomic and morphological or chemical constituents are needed’ (**See Lines 34-36 of the revised manuscript with track changes**)

**Comment #3.7.** L49-50-The flow is not smooth. I suggest that the information on chemical diversity is moved to after paragraph 73-81

**Response #3.7.** This was corrected (**See Lines 83-88 of the revised manuscript with track changes**).

**Comment #3.8.** L103-104 provide a citation

**Response #3.8.** The citation was provided (**Line 128 of the revised manuscript with track changes**).

**Comment #3.9.** L118-add ‘s’ to marker

**Response #3.9.** This was corrected (**Line 144 of the revised manuscript with track changes**)

**Comment #3.10.** L120-add ‘s’ to marker. They are SNP markers

**Response #3.10**. This was corrected (**Line 147 of the revised manuscript with track changes**)

**Comment #3.11**. L121-change essays to assays

**Response #3.11.** Change made (**Line 148 of the revised manuscript with track changes** )

**Comment #3.12.** L128-add ‘s’ to marker

**Response #3.12.** ‘s’ added to marker (**Line 155 of the revised manuscript with track changes**))

**Comment #3.13.** L129-instead put a comma after furthermore

**Response #3.13.** comma put after furthermore (**Line 155 of the revised manuscript with track changes**)Line 149)

**Comment #3.14.** L133-add ‘s’ to marker and check throughout the document

**Response #3.14.** This was corrected (**Line 159 of the revised manuscript with track changes**). In addition, this was checked throughout the document.

**Comment #3.15.** L138-139- edit the statement for clarity

**Response #3.15.** The statement was revised for clarity (**Lines 163-167 of the revised manuscript with track changes**))

“Despite the widespread use of single nucleotide polymorphisms (SNPs) in genetic studies, no research has yet reported the genetic diversity of *M. suaveolens* using SNP markers. This represents a significant gap that this study seeks to address. By generating SNP markers for *M. suaveolens,* we will make the first genetic sequences of this species accessible, providing a good foundation for further research on its evolutionary history, conservation, genetic mechanisms regulating essential oil yield and quality, and breeding purposes. The primary goal of this research is to generate molecular information that will support genetic improvement of *M. suaveolens* for enhanced essential oil yield and quality. Specifically, we aim to assess the extent of genetic diversity and population structure of *M. suaveolens* germplasm from Benin using high-density SNP markers. Comment #L159- please explain why you used 10 m. For most out-crossing species, 100 m is normally used to minimize sampling of similar individuals”.

**Comment #3.16.** Comment #L160-delete the second ‘the’

**Response #3.16.** the second ‘the’ was deleted (**Line 190 of the revised manuscript with track changes**)

**Comment #3.17.** L194-delete extra brackets after agarose

**Response #3.17.** The extra brackets were deleted after agarose This was corrected (**Line 226 of the revised manuscript with track changes**)

**Comment #3.18.** L261-265-I suggest that you use a Venn diagram for easy comprehension

**Response #3.18.** We did not perform a pairwise population comparison but we compared one population to the rest of populations. Therefore, using Venn diagram will not reflect the outcomes of the comparison.

**Comment #3.19.** L283-285. Overall, there is virtually no genetic differentiation. I hope this is well reflected in the discussion.

**Response #3.19.** We highlighted that the differentiation was very low. (**Line 438-534 of the revised manuscript with track changes**)

**Comment #3.20.** L332-342 has already been presented in the background. I suggest that you delete

**Response #3.20.** The paragraph was deleted **(Line 365-370 and 377-380 of the revised manuscript with track changes**).

**Comment #3.21.** L412-413 is misleading since there is virtually no genetic differentiation among all the 3 populations

**Response #3.22**. The phrase has been deleted (**Line 484-485 of the revised manuscript with track changes**)

**Comment #3.22.** L453-454. The low genetic differentiation could indicate high gene flow. Please explain how cross pollination can be helpful in enhancing diversity in this case.

**Response #3.22.** Gene flow can increase or decrease genetic diversity. When a population shows low genetic differentiation, it often indicates that genes are freely exchanged between individuals, probably through mechanisms such as cross-pollination. Cross-pollination increases genetic diversity in several ways: introducing new alleles and increasing variation. In this case, cross-pollination could enhance the genetic diversity by crossing the studied populations with another genetically distant population. That is why we suggested introducing new germplasms from different continents and countries would be essential steps toward increasing the genetic diversity of *M. suaveolens*. (**See Lines 525-534 of the revised manuscript with track changes**)

**Comment #3.23.** The conclusion should be re-written to highlight the major take-home messages, not just repeating the results.

**Response #3.23.**The conclusion was revised (**see Line 536-561 of the revised manuscript with track changes**).
