# Supplementary material for: SNP markers revealed the genetic diversity and population structure of Mesosphaerum suaveolens (L.) Kuntze Syn. Hyptis suaveolens (L.) Poit accessions collected in Benin
Source: PLoS One. 2025 Sep 4;20(9):e0331702. doi: 10.1371/journal.pone.0331702 (PMC12410747; doi:10.1371/journal.pone.0331702)
Supplement: Attachment — Submitted filename: Response to reviewers..docx [file pone.0331702.s008.docx]

**Manuscript ID: PONE-D-24-37428**

**Journal: PLOS ONE**

**Article type: Research Article**

**SNP markers revealed the genetic diversity and population structure of *Mesosphaerum suaveolens* (L.) Kuntze Syn. *Hyptis suaveolens* (L.) Poit accessions collected in Benin**

Armel Frida Dossa*, Dèdéou A. Tchokponhoué, Aristide Carlos Houdegbe, Enoch G. Achigan-Dako*

**Responses to reviewers' comments**

We thank the editor and the reviewer for their comments. We provided below a point-by-point response to their comments.

**Reviewer #3**

**Comment #3.1.** For consistence, I suggest you use both ‘improvement and conservation’ on L18 (mentions only improvement) since L31-32 use both terms.

**Response #3.1.** We thank the reviewer for this recommendation. Conservation was added to improvement (**See line 18** **of the revised manuscript with track changes**).

**Comment #3.2**. L40-41 revise to ‘subtropical climate of America’

**Response #3.2.** The correction was made as recommended. Please see **lines 40-41** **of the revised manuscript with track changes**.

**Comment #3.3.** L42- ‘perennial’ is more commonly used than pluriannual. You many consider to change

**Response #3.3.** Pluriannual was replaced by perennial. **(See line 42 of the revised manuscript with track changes)**

**Comment #3.4.** L46-Need to change the order the words from ‘xenogamy and geitonogamy’ to ‘respectively geitonogamy and xenogamy’. Geitonogamy involves pollination between flowers on the same plant (essentially self-pollination), while xenogamy involves pollination between flowers on different plants (cross pollination).

**Response #3.4.** The order of the words was changed from ‘xenogamy and geitonogamy’ to ‘geitonogamy and xenogamy’. (**Lines 46-47 of the revised manuscript with track changes**).

**Comment #3.5.** For better flow, you may need to move L44 to the end of the paragraph.

**Response #3.5.** The Line 44 was moved to the end of the paragraph as recommended **(see lines 48-49 of the revised manuscript with track changes)**.

**Comment #3.6.** Paragraph 1 and 2 are disjointed. I suggest you add a line at the end of paragraph 1 on the importance of the species-mentioning the essential oils as key, to lead the readers to paragraph 2.

**Response #3.6.** A sentence was added at the end of paragraph 1 (**See lines 50-51 of the revised manuscript with track changes**)

**Comment #3.7.** Table 1: The average of Fis is different from what you indicated in L235, please clarify.

**Response #3.7.** Thank you for the comment. Sorry, the average value of Fis was misreported. The true value was 0.592 and now corrected (**See Line 243 of the revised manuscript with track changes**). However, the average values of Fis presented in Table 1 are for each studied population. Therefore, to avoid misunderstanding the overall average values of the diversity statistics across all populations, including the Fis were added in Table 1.

**Comment #3.8.** L243-244 I don’t think the allelic richness among the populations is different. A difference of 0.001 or 0.006 may not be statistically significant. Consider revising.

**Response #3.8.** True that the difference was not statistically significant. This is now clearly indicated in the sentence that now reads: “Although the mean allelic richness seemed not statistically different among population, the Guinean population presented the highest value, whereas the Sudano-guinean population presented the lowest value for this parameter.” **(Lines 250-251 of the revised manuscript with track changes**)

**Comment #3.9.** L251-253 and the table are a bit hard to follow. Consider simplifying them if possible.

**Response #3.9.** The sentences were improved (**Lines 260-264 of the revised manuscript with track changes**). The table was improved too (**Table 2 of the revised manuscript with track changes**).

**Comment #3.10.** On L350, Fis = 0.59 and is different from the Fis presented in L235 (Fis=0.289), please clarify

**Response #3.10**. The average value of Fis in (L235) was misreported. The true values was Fis = 0.59 and was corrected accordingly (**See Line 243 of the revised manuscript with track changes**).
